# A fluorescent sensor for real-time measurement of extracellular oxytocin dynamics in the brain

**DOI:** 10.1038/s41592-022-01597-x

**Published:** 2022-09-22

**Authors:** Daisuke Ino, Yudai Tanaka, Hiroshi Hibino, Masaaki Nishiyama

**Affiliations:** 1 Department of Histology and Cell Biology, Graduate School of Medical Sciences, Kanazawa University, Kanazawa, Japan; 2 Department of Pharmacology, Graduate School of Medicine, Osaka University, Osaka, Japan; 3 Department of Molecular and Cellular Pathology, Graduate School of Medical Sciences, Kanazawa University, Kanazawa, Japan

**Keywords:** Synaptic transmission, Neurophysiology, Mouse, Fluorescent proteins

## Abstract

Oxytocin (OT), a hypothalamic neuropeptide that acts as a neuromodulator in the brain, orchestrates a variety of animal behaviors. However, the relationship between brain OT dynamics and complex animal behaviors remains largely elusive, partly because of the lack of a suitable technique for its real-time recording in vivo. Here, we describe MTRIA_OT_, a G-protein-coupled receptor-based green fluorescent OT sensor that has a large dynamic range, suitable affinity, ligand specificity for OT orthologs, minimal effects on downstream signaling and long-term fluorescence stability. By combining viral gene delivery and fiber photometry-mediated fluorescence measurements, we demonstrate the utility of MTRIA_OT_ for real-time detection of brain OT dynamics in living mice. MTRIA_OT_-mediated measurements indicate variability of OT dynamics depending on the behavioral context and physical condition of an animal. MTRIA_OT_ will likely enable the analysis of OT dynamics in a variety of physiological and pathological processes.

## Abstract (web summary)

A fluorescent sensor for oxytocin called MTRIA_OT_ has been developed. The sensor’s capabilities are demonstrated in fiber photometry measurements in freely behaving mice.

## Main

OT, a neuropeptide produced by neurons in the paraventricular nucleus (PVN) and supraoptic nucleus of the hypothalamus, regulates many physiological processes in both peripheral tissues and the central nervous system. OT neurons primarily send axons to the posterior pituitary, where OT is released into the peripheral circulation as a peripheral hormone that promotes childbirth and lactation. In addition, axons of OT neurons project to numerous other brain regions, where they release OT to regulate diverse physiological functions, such as sensory processing, feeding control, social cognition and emotion^1^. Impairment of OT signaling in the brain may underlie cognitive and emotional dysfunction associated with neurodevelopmental disorders (for example, autism spectrum disorders and schizophrenia) and brain aging^2,3^. Given that the timescales of OT-related physiological and pathological phenomena are diverse (from seconds, minutes, hours and days to potentially much longer periods), the dynamics of OT in the brain may be variable depending on behavioral patterns, types of stimuli and physical conditions of animals.

In addition to its roles as an endogenous ligand, OT has emerged as a potential therapeutic agent for psychiatric disorders based on the finding that administration of exogenous OT enhances positive emotions in humans^4^. Therefore, OT administered peripherally, such as via intranasal or intravenous routes, was originally believed to reach the brain and exert therapeutic effects. However, the potency of exogenous OT administration is contraversial^5–7^, and a recent study in humans mostly invalidated initial observations of the effects of exogenous OT administration^8^. Therefore, whether OT administered via a peripheral route can efficiently reach the brain through the nose–brain pathway and/or blood–brain barrier remains unclear^5^.

In this context, techniques permitting detection of brain OT dynamics are needed. However, currently available methods, such as microdialysis^9–11^ and a reporter gene-based assay (that is, iTango)^12^, have limitations, especially in terms of temporal resolution. Recently, fluorescent sensors consisting of a G-protein-coupled receptor (GPCR) with a fluorescent protein replacing the amino acids in its third intracellular loop (IL3), have been engineered as promising tools for real-time detection of neurotransmitters and neuromodulators such as dopamine, acetylcholine, norepinephrine and adenosine^13–18^. Inspired by these strategies, we developed a sensitive fluorescent OT sensor named MTRIA_OT_, which is composed of a medaka OT receptor (OTR) and a circularly permutated green fluorescent protein (cpGFP)-based fluorescent module named MTRIA (*M*odular fluorescence unit fused with *TR*ansmembrane region-to-*I*ntr*A*cellular loop linkers). Using fiber photometry-mediated fluorescence recording, we demonstrate that MTRIA_OT_ can report a variety of OT dynamics in the mouse brain, including artificially evoked OT signals, endogenous OT responses during natural behaviors and altered endogenous OT dynamics by chemical and physical perturbations.

## Results

### Development of a sensitive fluorescent oxytocin sensor

**Fig. 1:**
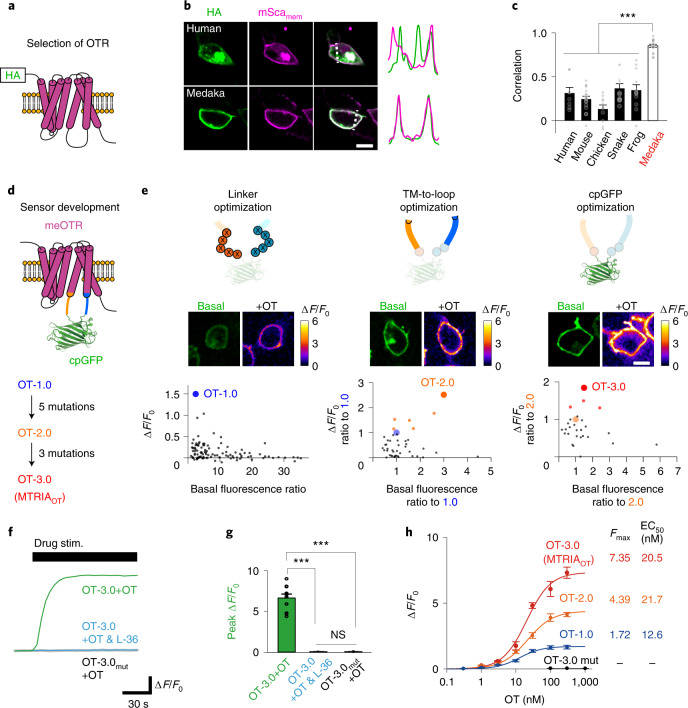
Development of a fluorescent oxytocin sensor. **a**, Schematic of a PM-localized OTR conjugated with an HA-tag at the N terminus. **b**, Images of HEK293T cells coexpressing an HA-tagged OTR (HA, green) and PM-targeted mScarlet (mSca_mem_, magenta). Traces on right compare the normalized fluorescence intensities of HA and mSca_mem_ signals along the dotted lines. **c**, Summary of Pearson correlation coefficients of fluorescence signals in **b** (*n* = 8, 17, 10, 10, 12 and 17 cells for human, mouse, chicken, snake, frog and medaka, respectively). Statistics used were one-way analysis of variance (ANOVA; *F*_5,68_ = 2.35, *P* = 4.9 × 10^−19^) with Bonferroni post hoc test (*P* = 3.3 × 10^−9^, human versus medaka; *P* = 8.9 × 10^−9^, mouse versus medaka; *P* = 5.4 × 10^−14^, chicken versus medaka; *P* = 2.4 × 10^−19^, snake versus medaka; *P* = 2.3 × 10^−8^, frog versus medaka). **d**, Schematic of the sensor architecture. **e**, Development of a sensitive fluorescent OT sensor over a three-step screening; optimization of linker regions, TM-to-loop region and cpGFP moiety. Schematics of mutagenesis (top), basal fluorescence images and heat maps depicting responses to 100 nM OT (middle), and scatterplots describing the relationship between basal brightness and the fluorescence response to 100 nM OT (bottom). **f**, Representative traces of fluorescence responses upon simulation with the indicated drug (green, OT-3.0-expressing cells upon stimulation with OT; cyan, OT-3.0-expressing cells upon stimulation with a mixture of OT and L-36; gray, OT-3.0-mut-expressing cells upon stimulation with OT). **g**, Summary of peak Δ*F*/*F*_0_ values (*n* = 10 cells per group). Statistics used were one-way ANOVA (*F*_2,27_ = 3.35, *P* = 5.7 × 10^−16^) with Bonferroni post hoc test (*P* = 4.3 × 10^−10^, left versus middle; *P* = 4.3 × 10^−10^, left versus right; *P* = 1, middle versus right). **h**, Dose–response curves of sensors (*n* = 10 cells per point). *F*_max_ and EC_50_ values are summarized on the right. Scale bars, 10 µm (**b** and **e**). Graphs represent the mean ± s.e.m. (**c**, **g** and **h**). ***P < 0.001, NS, not significant (**c** and **g**). Source data

**Extended Data Fig. 1:**
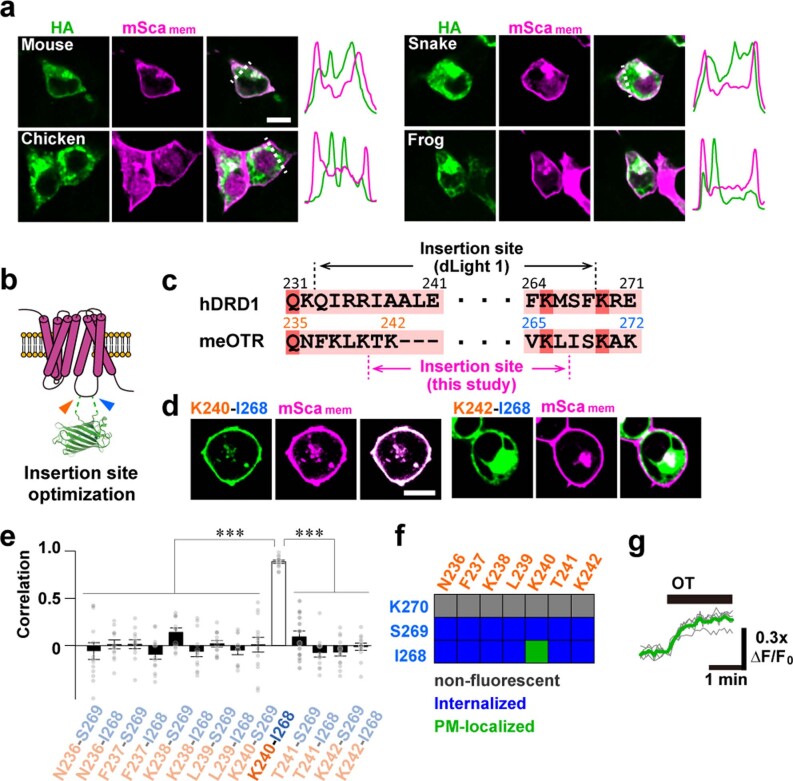
Optimization of a fluorescent OT sensor. **a**, Representative images of HEK293T cells co-expressing an HA-tagged OTR (green) and a mSca_mem_ (magenta). Normalized fluorescence intensities on the dotted lines are shown on the right. **b**, Schematic representation of cpGFP insertion into the IL3 of OTR. **c**, Sequence alignment of regions of the IL3 of human dopamine receptor D1 (hDRD1), a scaffold of a dopamine sensor (dLight1), and meOTR. **d**, Representative images of HEK293T cells co-expressing the indicated meOTR-cpGFP chimera (green) and mSca_mem_ (magenta). **e**, Pearson correlation coefficients comparing meOTR-cpGFP chimeras and mSca_mem_ are summarized as mean ± SEM (*n* = 13, 12, 12, 13, 12, 13, 13, 11, 13, 13, 13, 12, 13, and 12 cells; left to right). Statistics: one-way ANOVA (*F*_13,167_ = 1.78, *P* = 5.5 × 10^−32^) with Bonferroni *post-hoc* test (*P* = 5.1 × 10^−9^: N236–S269, *P* = 7.9 × 10^−14^: N236–I268, *P* = 1.2 × 10^−13^: F237–S269, *P* = 4.1 × 10^−14^: F237–I268, *P* = 3.1 × 10^−12^: K238–S269, *P* = 4.9 × 10^−14^: K238–I268, *P* = 1.6 × 10^−16^: L239–S269, *P* = 2.9 × 10^−14^: L239–I268, *P* = 1.2 × 10^−9^: K240–S269, *P* = 9.6 × 10^−11^: T241–S269, *P* = 5.4 × 10^−15^: T241–I268, *P* = 2.7 × 10^−16^: K242–S269, *P* = 2.0 × 10^−16^: K242–I268, compared with K240–I268). ***P < 0.001. **f**, Summary of insertion site-dependent characteristics of meOTR-cpGFP chimeras. **g**, Traces showing fluorescence responses of the K240–I268 meOTR-cpGFP chimera upon stimulation with 100 nM OT. Scale bars, 10 µm (**a**, **d**). Source data

**Extended Data Fig. 2:**
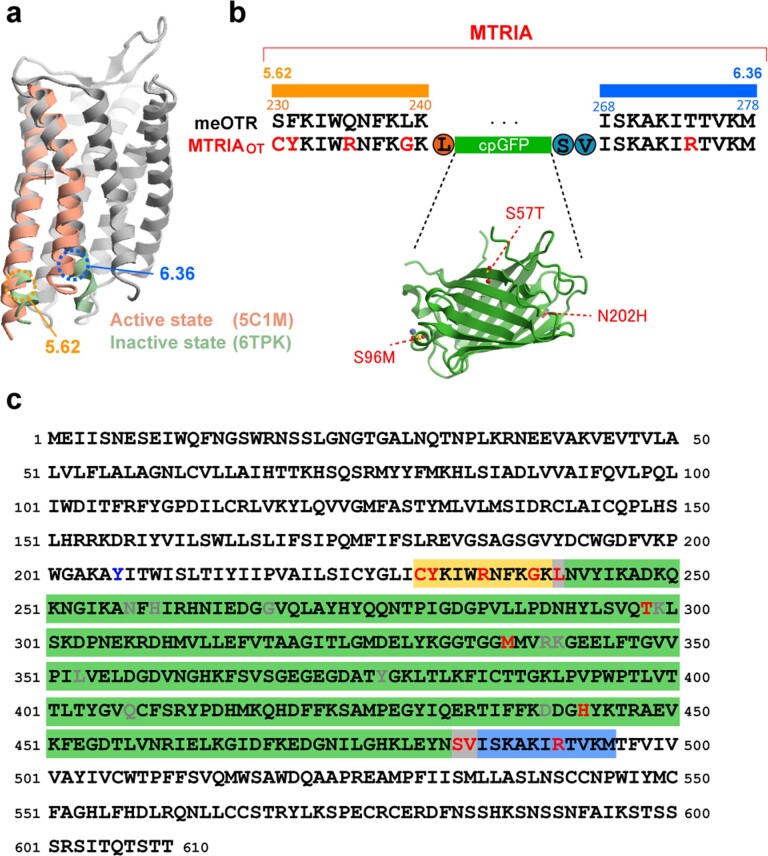
Sequence of our fluorescent OT sensor. **a**, Superposition of active- and inactive-GPCR structures, adapted from the Protein Data Bank (PDB) archives (IDs: 5C1M and 6TPK). TM5–TM6 regions of active and inactive states are colored pink and green, respectively. **b**, Alignment of the TM5–TM6 region between meOTR and MTRIA_OT_; the structure of cpGFP is adapted from a PDB archive (ID: 3SG2). Mutations introduced in MTRIA_OT_ are shown as red. **c**, Full amino acid sequence of MTRIA_OT_. Mutations in MTRIA_OT_, the point mutation in MTRIA_OT_-mut, and mutations of cpGFP that were adapted in the other fluorescent sensors are shown in red, blue, and gray, respectively.

We initially conducted a preliminary screen for an OTR that showed good targeting to the plasma membrane (PM), a key property for PM-targeted fluorescent sensors^15,19,20^. We chose medaka OTR (meOTR) as a scaffold for our fluorescent sensor because this receptor showed the best targeting to the PM among candidate OTRs from six vertebrate species (human, mouse, chicken, snake, frog and medaka) in human embryonic kidney 293T (HEK293T) cells (Fig. 1a–c and Extended Data Fig. 1a). After optimization of the insertion site of cpGFP in the IL3 of meOTR (Extended Data Fig. 1b–g and Supplementary Note 1), we screened the mutant sensors in HEK293T cells using the following three steps (Fig. 1d and Supplementary Note 1). We sequentially performed mutagenesis in linkers in the N-terminal and C-terminal regions surrounding the cpGFP, neighboring regions of the linkers ranging from the transmembrane helix to the intracellular loops (TM-to-loop) and residues in cpGFP (Fig. 1e). Through these three screening steps, we obtained OT-1.0, OT-2.0 and OT-3.0, respectively. OT-3.0, the final product of the screening, had an approximately 720% Δ*F*/*F*_0_ fluorescence response upon stimulation with 100 nM OT, which was suppressed by the OTR antagonist L-368,899 (L-36) (Fig. 1f,g). We also generated an OT-insensitive sensor (OT-3.0-mut) that contained the Y206A mutation, which abolished its ligand-binding capacity^21^ (Fig. 1f,g). Characterization of the dose-dependent fluorescence responses of OT-1.0 to OT-3.0 in HEK293T cells (Fig. 1h) showed that our screening improved the dynamic range of fluorescence responses (*F*_max_) with little change to the half-maximal effective concentration (EC_50_) value. We designated the fluorescent module of OT-3.0 as MTRIA (Extended Data Fig. 2) and renamed OT-3.0 as MTRIA_OT_.

**Extended Data Fig. 3:**
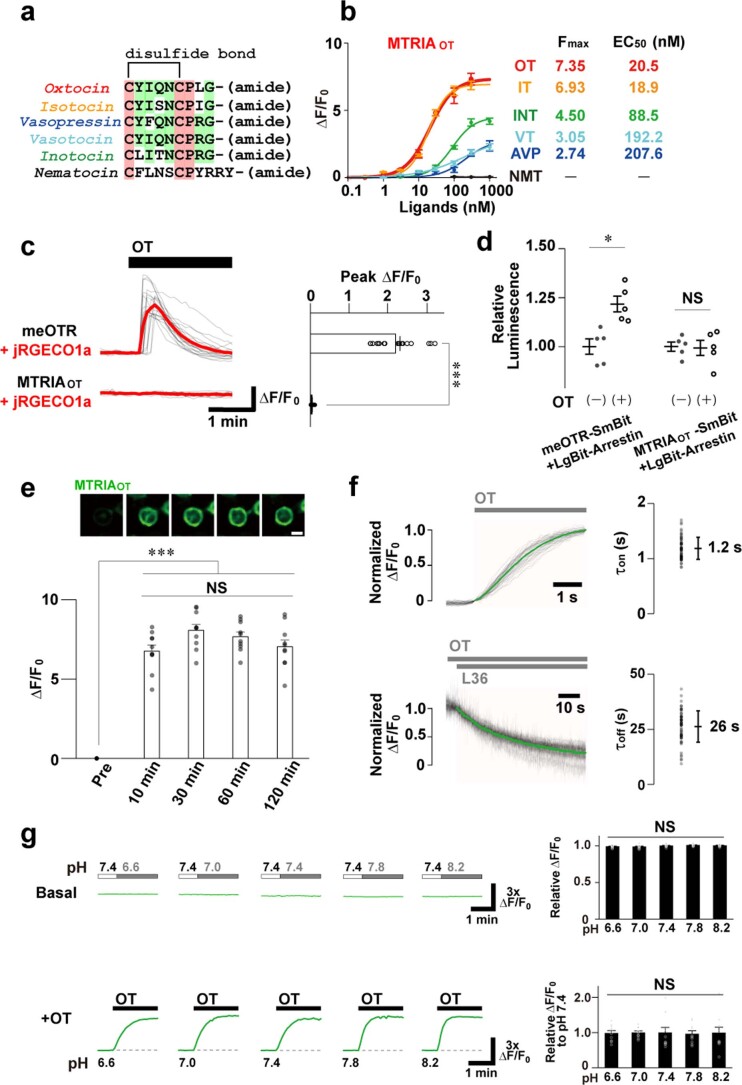
The basic properties of MTRIA_OT_ in HEK293T cells. **a**, Sequence alignment of OT and its orthologous neuropeptides. **b**, Dose-response curves of MTRIA_OT_ to OT and its orthologous neuropeptides (*n* = 10 cells per point). **c**, Assay for G protein coupling. Traces of jRGECO1a fluorescence responses in either meOTR- or MTRIA_OT_-co-expressing cells upon stimulation with 100 nM OT (gray: each trace, red: average trace). Summary of peak Δ*F*/*F*_0_ responses are shown on the right (*n* = 20 cells). **d**, Assay for β-arrestin coupling. Traces showing the relative luminescence increase induced by NanoLuc luciferase complementation upon stimulation with 100 nM OT (*n* = 5 wells). **e**, Time-course of the fluorescence intensity of MTRIA_OT_ over 120 min following 100 nM OT stimulation. Representative images (top) and summary of Δ*F*/*F*_0_ responses (bottom: *n* = 10 cells). **f**, Traces showing the kinetics of MTRIA_OT_ in the fluorescence rise (top left: τ_on_) upon 5 μM OT and decay (bottom left: τ_off_) upon 300 nM OT and subsequent 10 μM L36 (gray: each trace, green: average trace; *n* = 60 cells). The time constants determined by a single-exponential fitting are summarized to the right. **g**, Representative traces showing the dependence of MTRIA_OT_ fluorescence intensity on extracellular pH (left top: basal fluorescence levels, bottom: OT-induced responses). Summary of basal fluorescence levels normalized by the value just before pH change (right top: *n* = 10 cells) and OT-induced responses formalized by the mean of Δ*F*/*F*_0_ at pH 7.4 (right bottom: *n* = 10 cells). Scale bar, 10 µm (**e**). Graphs represent mean ± SEM (**b–e, g**) and mean ± SD (**f**). ***P < 0.001, *P < 0.05, NS: not significant (**c–e, g**). Statistics (**b**, **d**, **e**, **g)** are summarized in Supplementary Note 4. Source data

We then further characterized the ligand specificity of MTRIA_OT_. Compared to its sensitivity to OT, the sensor was similarly sensitive to isotocin (an OT analog in fish), much less sensitive to vasopressin orthologs (vasopressin and vasotocin) and inotocin (an OT and vasopressin ortholog in insects), and insensitive to nematocin (an OT and vasopressin ortholog in nematodes; Extended Data Fig. 3a,b). We also examined the basic properties of MTRIA_OT_, such as its coupling capacity with downstream effectors, long-term fluorescence stability, kinetics and pH sensitivity. We used a Ca^2+^ imaging experiment and a split-luciferase complementation assay to assess the coupling of MTRIA_OT_ with G_αq_ protein and β-arrestin signaling pathways. MTRIA_OT_ had no detectable effects on either of these effectors, whereas wild-type meOTR was able to couple with them (Extended Data Fig. 3c,d). Furthermore, we did not detect internalization of MTRIA_OT_ from the PM when we chronically exposed MTRIA_OT_-expressing HEK293T cells to 100 nM OT (Extended Data Fig. 3e). We conducted a kinetic analysis of MTRIA_OT_ using a local puff of OT and L-36, which yielded an on-rate and an off-rate of approximately 1.2 s and 26 s, respectively (Extended Data Fig. 3f). Finally, changes in the extracellular pH level ranging from 6.6 to 8.2 minimally affected basal and OT-induced MTRIA_OT_ fluorescence (Extended Data Fig. 3g), indicating little pH dependence of MTRIA_OT_ within the physiological extracellular pH range.

**Extended Data Fig. 4:**
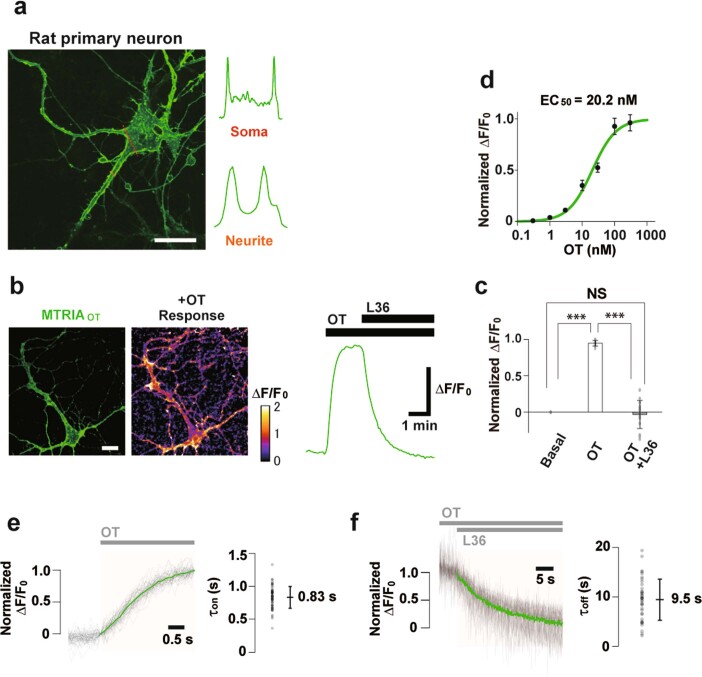
The basic properties of MTRIA_OT_ in rat hippocampal primary neurons. **a**, Representative image showing a rat primary neuron expressing MTRIA_OT_ and profiles of fluorescence intensities along the dotted lines (red: soma, orange: neurite). **b**, Fluorescence image (left), heatmap image depicting the response to 300 nM OT (middle), and representative trace of MTRIA_OT_ response upon 300 nM OT and subsequent 1 μM L36 (right). **c**, Summary of Δ*F*/*F*_0_ responses normalized by a peak value during OT stimulation (*n* = 15 cells). Statistics: one-way ANOVA (*F*_2, 42_ = 3.22, *P* = 3.5 × 10^−27^) with Bonferroni *post-hoc* test (*P* = 1.0 × 10^−20^: Basal vs. OT, *P* = 8.1 × 10^−11^: OT vs. OT + L36, *P* = 1: Basal vs. OT + L36). **d**, Dose-response curves of MTRIA_OT_ in primary neurons (*n* = 18, 24, 26, 18, 15, 35, and 42 cells for 0.3, 1, 3, 10, 30, 100, and 300 nM, respectively). Values were normalized against a maximum value of the fitted-sigmoidal curve. **e–f**, Traces showing the kinetics of MTRIA_OT_ in the fluorescence rise (**e** left: τ_on_) upon 5 μM OT and decay (**f** left: τ_off_) upon 300 nM OT and subsequent 1 μM L36 (gray: each trace, green: average trace; *n* = 59 cells in **e** and 38 cells in **f**). Time constants determined by a single-exponential fitting are summarized to the right. Scale bars, 20 µm (**a**) and 30 µm (**b**). Graphs represent mean ± SEM (**c**, **d**) and mean ± SD (**e**, **f**). ***P < 0.001, NS: not significant (**c**). Source data

We also examined the performance of MTRIA_OT_ in cultured rat hippocampal neurons. When expressed in primary neurons, MTRIA_OT_ showed good PM localization both in soma and neurites (Extended Data Fig. 4a). Application of OT induced a robust increase in the fluorescence intensity of MTRIA_OT_, and subsequent application of L-36 suppressed its fluorescence to a level comparable to basal fluorescence (Extended Data Fig. 4b,c). The EC_50_ value of MTRIA_OT_ measured in primary neurons was 20.2 nM (Extended Data Fig. 4d), almost the same value as that measured in HEK293T cells. Kinetic time constants measured in primary neurons were faster (on-rate of ~0.83 s, off-rate of ~9.5 s; Extended Data Fig. 4e,f) than those observed in HEK293T cells, similar to a previously reported GPCR-based fluorescent sensor^18^. Taken together, these results indicate that our MTRIA_OT_ sensor is equipped with enough sensitivity, specificity and stability to accurately detect extracellular OT dynamics.

### Validation of MTRIA_OT_ in vivo

**Fig. 2:**
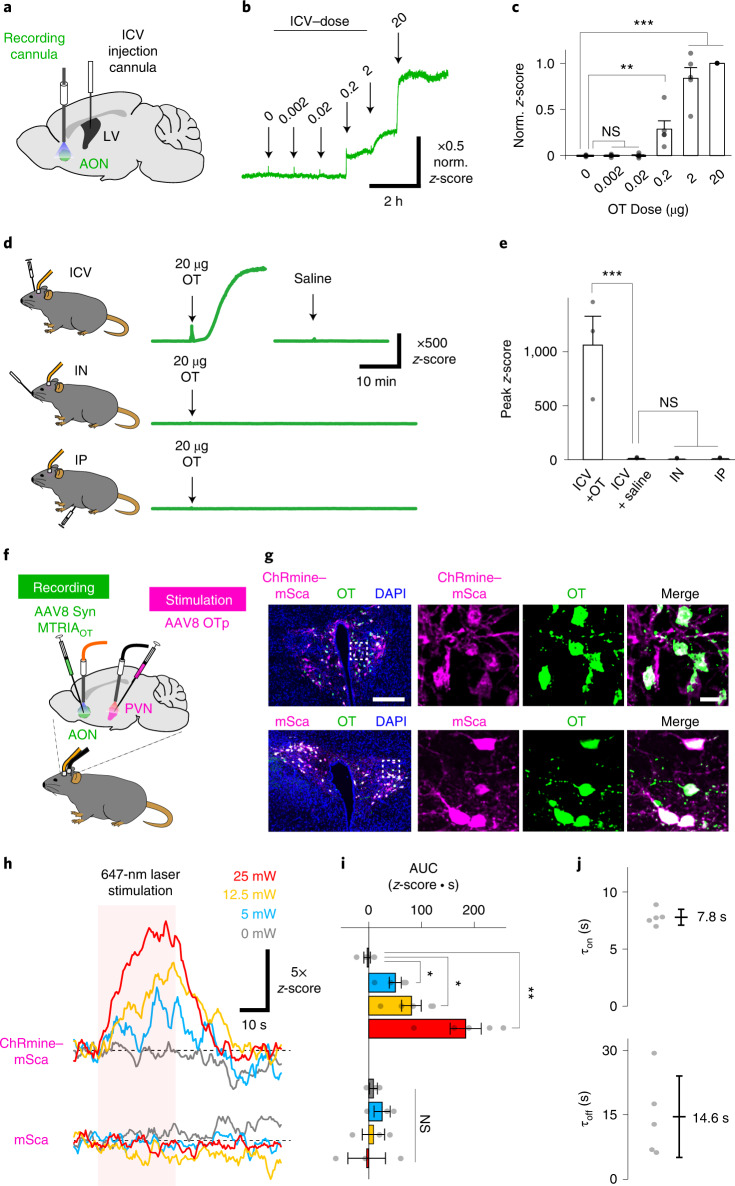
In vivo real-time measurement of brain oxytocin dynamics following exogenous oxytocin administration and optogenetic stimulation of oxytocin neurons. **a**, Schematic illustrating fiber photometry recording of MTRIA_OT_ in the AON. **b**, Representative trace of the *z*-scored fluorescence intensity of MTRIA_OT_ following intracerebroventricular (ICV) injection of OT at the indicated doses. The amplitude of the signal is shown as a value normalized against the peak value of the highest dose (norm. *z*-score). **c**, Summary of normalized *z*-score (*n* = 5 mice). **d**, Representative traces of *z*-scores upon stimulation with either 20 µg OT or saline via the indicated administration routes (ICV, intranasal (IN) and intraperitoneal (IP)). **e**, Summary of peak *z*-scores (*n* = 3 mice). **f**, Schematic illustrating fiber photometry recording of MTRIA_OT_ in the AON upon optogenetic stimulation of OT neurons in the PVN. **g**, Histological images showing transduction of the indicated gene products (ChRmine–mSca and mSca) in OT-positive neurons in the PVN. Overlaid images of the indicated gene product (magenta), OT staining (green) and DAPI staining (blue) are shown to the left. Magnified images within the dashed rectangles are shown on the right. **h**, Representative traces of MTRIA_OT_ responses to the light stimuli at the indicated powers recorded in a mouse expressing either ChRmine–mSca or mSca in OT neurons. The period of light stimulation is indicated by the pink-shaded area. **i**, Summary of area under the curve (AUC) values during light stimulation (*n* = 5 mice in ChRmine–mSca, *n* = 3 mice in mSca). **j**, Summary of rise and decay time constants (*n* = 5 mice). Scale bars, 200 µm (left) and 20 µm (right) in **g**. Graphs represent the mean ± s.e.m. (**c**, **e** and **i**) and the mean ± s.d. (**j**). ***P < 0.001, **P < 0.01, *P < 0.05, NS (**c**, **e** and **i**). Statistics (**c**, **e** and **i**) are summarized in Supplementary Note 2. Source data

**Extended Data Fig. 5:**
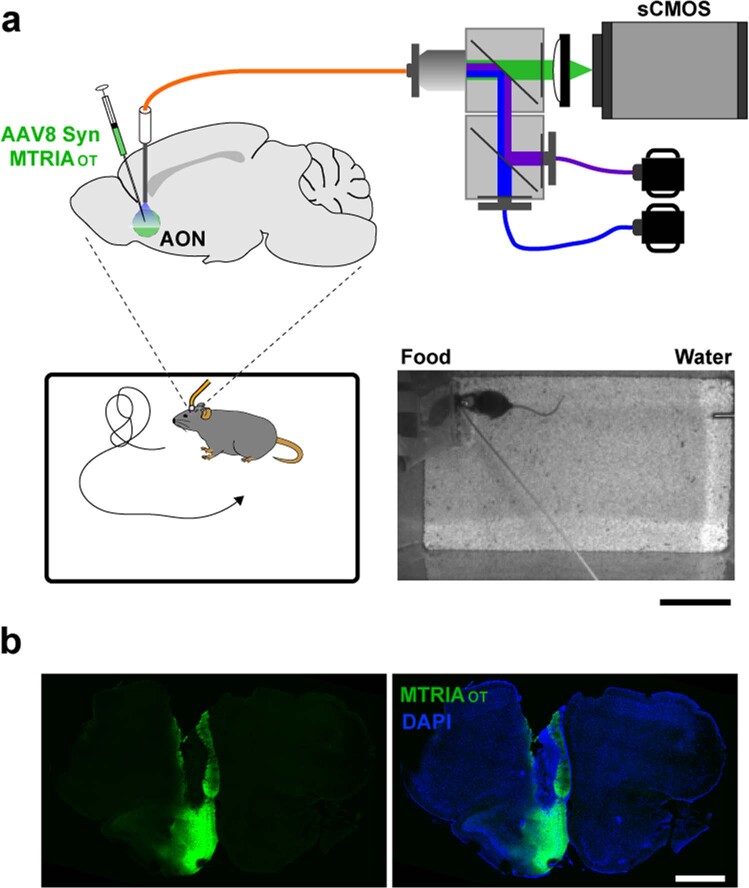
Fiber photometry setup and confirmation of the measurement site. **a**, Schematic illustrating fiber photometry measurements in a freely behaving mouse. A representative image of an experimental arena captured by an overhead camera is shown in the bottom right. **b**, Histology showing expression of MTRIA_OT_ (green) and the placement of an implanted cannula. DAPI (blue) was used for counterstaining nuclei. Scale bars, 10 cm (**a**) and 1 mm (**b**).

Having validated the basic properties of MTRIA_OT_ in cultured cells, we next tested whether our OT sensor is applicable to experiments in the brains of living mice. Using an adeno-associated virus (AAV), we expressed MTRIA_OT_ in the anterior olfactory nucleus (AON), a major target site of OT in the brain with high OTR expression levels^22,23^. We then conducted fiber photometry recordings through an implanted cannula placed above the injection site (Fig. 2a and Extended Data Fig. 5). First, we examined the responses of MTRIA_OT_ after intracerebroventricular infusion of 10 µl saline containing various amounts of OT (0, 0.002, 0.02, 0.2, 2 and 20 µg) in anesthetized mice. When we serially applied increasing amounts of OT through a stainless-steel cannula implanted into the lateral ventricle (Fig. 2a), we observed robust fluorescence increases upon stimulation with OT at doses of 0.2 µg and above (Fig. 2b,c), comparable doses to those which are required to trigger OT-dependent animal behaviors^24–26^. This finding indicates that MTRIA_OT_ is capable of real-time detection of extracellular OT in living brains.

Peripheral administration of less than 20 µg of OT is reportedly sufficient to affect animal behaviors^27,28^; therefore, we next evaluated whether OT levels in the AON increased after application of exogenous OT from two distinct peripheral administration routes: intranasal and intraperitoneal. Neither intranasal nor intraperitoneal administration of 20 µg of OT induced detectable fluorescence responses of MTRIA_OT_ in anesthetized mice (Fig. 2d,e). Taken together with the finding that MTRIA_OT_ had robust responses to intracerebroventricular administration of OT solution at a dose of 0.2 µg (Fig. 2b,c), our data indicate that less than 1% of peripherally administered OT can reach the AON.

We next examined whether MTRIA_OT_ can detect optically evoked OT release by combining fiber photometry recording and optogenetic stimulation. To this end, we virally expressed ChRmine–mSca, a fusion protein of red-shifted channelrhodopsin ChRmine^29^ and mScarlet (mSca), in OT-expressing PVN neurons and MTRIA_OT_ in AON cells (Fig. 2f). For control experiments, we expressed mSca instead of ChRmine–mSca in OT neurons. Our histological analysis verified that AAV vectors harboring the OT promoter (OTp) resulted in the expression of gene products specifically in OT neurons in the PVN (Fig. 2g). We measured MTRIA_OT_ responses in the AON upon optogenetic stimulation of OT neurons with trains of red-light pulses (647-nm wavelength, 10-ms pulse width, 20-Hz frequency) lasting for 30 s, and parameters were comparable to those previously used to evoke optogenetic OT release^23,30,31^. We found that MTRIA_OT_ signals in the AON of ChRmine-expressing mice were progressively enhanced with increasing power of the stimulation laser (from 0, 5 and 12.5 to 25 mW) whereas those in control mice showed no detectable changes upon stimulation at any laser intensity (Fig. 2h,i). To estimate the kinetic properties of MTRIA_OT_ in vivo, we determined the time constants of OT signals induced with 25 mW of light, which were stable enough for a single-exponential fitting. The mean rise and decay times were 7.8 s and 14.6 s, respectively (Fig. 2j). Taken together, these observations indicate that MTRIA_OT_ can be used to measure endogenous OT release in vivo with high sensitivity and rapid kinetics.

### Monitoring oxytocin dynamics in the brains of freely behaving mice

**Fig. 3:**
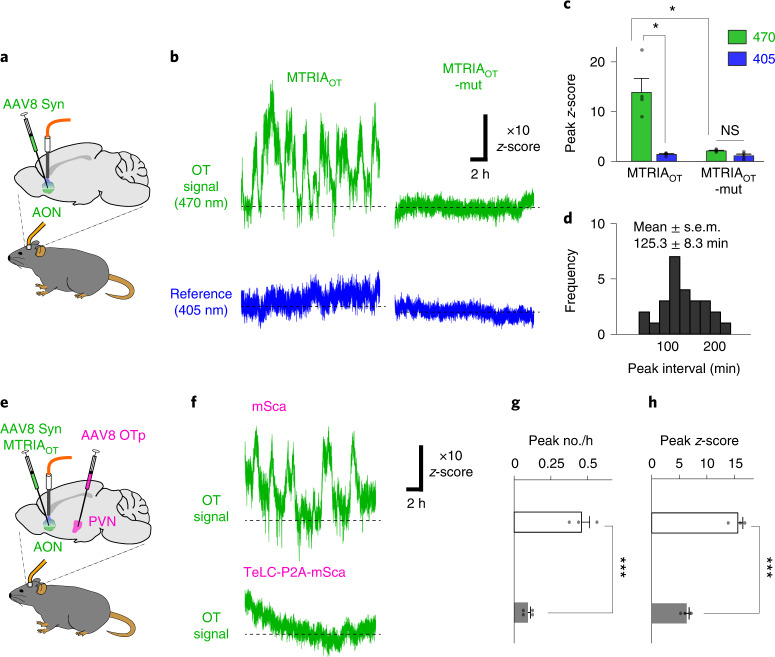
In vivo real-time measurement of brain oxytocin oscillation in freely behaving mice. **a**, Schematic illustrating fiber photometry recording of MTRIA_OT_ in the AON in freely behaving mice. **b**, Representative traces of 470-nm-excited signals (green) and 405-nm-excited signals (blue) from either MTRIA_OT_-expressing or MTRIA_OT_-mut-expressing mice. **c**, Summary of peak *z*-scores (*n* = 4 mice). Statistics used were one-way ANOVA (*F*_3,12_ = 3.49, *P* = 7.4 × 10^−5^) with Bonferroni post hoc test (*P* = 0.026, 470 nm versus 405 nm in MTRIA_OT_; *P* = 0.034, 470 nm in MTRIA_OT_ versus 470 nm in MTRIA_OT_-mut; *P* = 0.21, 470 nm versus 405 nm in MTRIA_OT_-mut). **d**, Frequency histogram showing the intervals of OT signal peaks (*n* = 26 events from four mice). **e**, Schematic illustrating experimental protocol for assessing the involvement of OT release from PVN neurons in OT signal increases in the AON. **f**, Representative traces of MTRIA_OT_ activities recorded from mice either expressing mSca or coexpressing TeLC and mSca in the PVN. **g**,**h**, Summary of the peak number every hour (**g**) and peak *z*-score (**h**; *n* = 3 mice in mSca and *n* = 4 mice in TeLC-P2A-mSca). Statistics used were an unpaired two-tailed *t*-test (*P* = 8.2 × 10^−4^ in **g** and *P* = 1.6 × 10^−4^ in **h**). Graphs represent the mean ± s.e.m. (**c**, **g** and **h**). ***P < 0.001, *P < 0.05, NS (**c**, **g** and **h**). Source data

**Extended Data Fig. 6:**
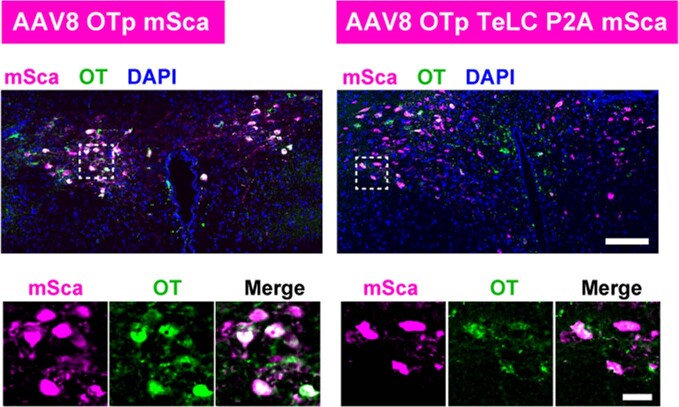
Histological verification of specific gene transduction in OT neurons. Histology showing transduction of the indicated AAV vectors (left: AAV8 OTp mSca; right: AAV8 OTp TeLC P2A mSca) in OT-positive neurons in the PVN. Overlaid images of mSca (magenta), OT staining (green), and DAPI staining (blue) are shown in the top panels. Magnified images within the dotted rectangles are shown in the bottom panels. Scale bars, 100 µm (top) and 20 µm (bottom).

Having shown that MTRIA_OT_ is functional in the mouse brain, we next examined whether MTRIA_OT_ can be used to assess endogenous OT dynamics in the brains of behaving mice. OT levels in cerebrospinal fluid reportedly show daily fluctuations^11,32^; however, the precise temporal profile has not been fully determined, largely because of the limited sampling rate of conventional approaches. Therefore, we recorded MTRIA_OT_ responses during daily behavior. We virally expressed MTRIA_OT_ in the AON and measured its fluorescence responses using fiber photometry (Fig. 3a). We found that transient increases of OT signals were repeated at approximately 2-h intervals in the AON of mice freely behaving in a cage with food and water supplied ad libitum (Fig. 3b–d). We named this ultradian OT rhythm ‘OT oscillation’. OT oscillations were absent in simultaneously recorded reference signals (405-nm excited MTRIA_OT_) and in signals recorded using control sensors (470-nm and 405-nm excited MTRIA_OT_-mut; Fig. 3b), excluding the involvement of artifacts derived from movements and/or autofluorescence. We selectively transduced tetanus toxin light chain (TeLC), which prevents vesicular transmitter release^33^, in OT-expressing neurons in the PVN using an AAV vector harboring the OT promoter (Extended Data Fig. 6). Expression of TeLC suppressed the increase in OT compared with the control (Fig. 3e–h), confirming that OT oscillation is dependent on OT release from oxytocinergic neurons in the PVN. These results demonstrate the utility of MTRIA_OT_ to capture ultradian OT dynamics in the brain.

**Fig. 4:**
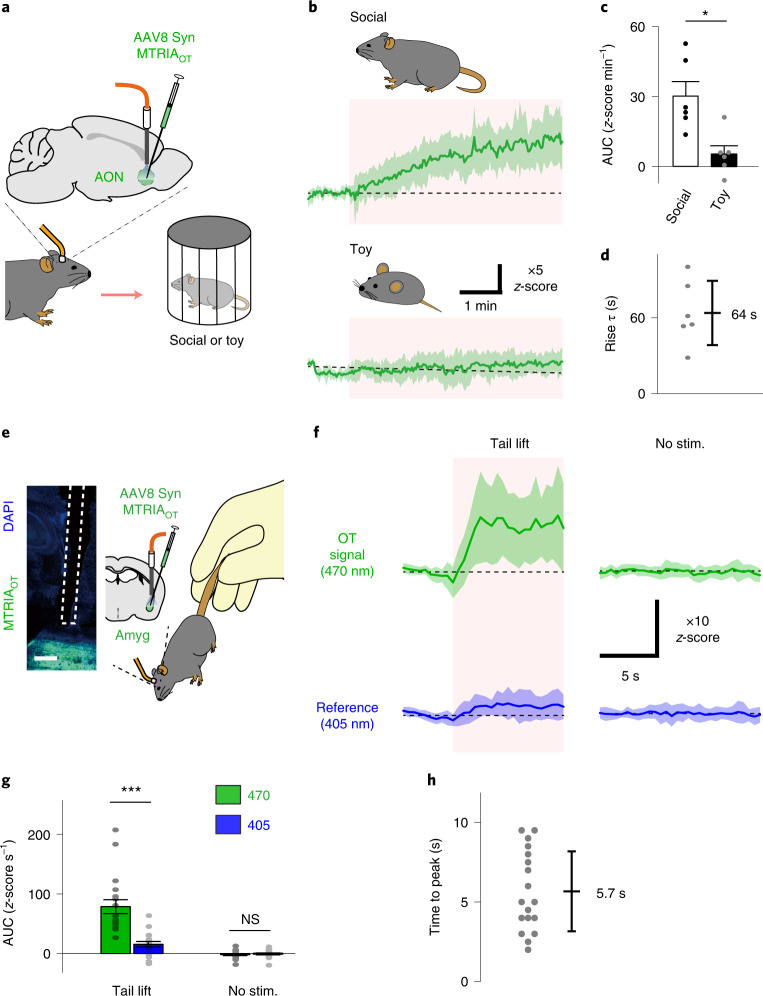
In vivo real-time measurement of brain oxytocin responses upon social interaction and acute stress. **a**, Schematic illustrating fiber photometry recording of social interaction-induced OT responses. **b**, Mean *z*-scored traces (green thick lines) showing MTRIA_OT_ responses to encounters with either a mouse (top) or a toy (bottom). The regions covering s.d. values of the traces are shaded in light green. Stimulation was applied during the periods shaded in pink. **c**, Summary of AUC values during stimulation (*n* = 6 mice). Statistics used were a paired two-tailed *t*-test (*P* = 0.03). **d**, Summary of rise time constants for social interaction-induced OT responses determined by a single-exponential fitting (*n* = 6 mice). **e**, Histological verification of sensor expression around the recording region (left) and schematic illustrating fiber photometry recording of social interaction-induced OT responses (right). **f**, Mean *z*-scored traces showing MTRIA_OT_ responses (green, 470-nm-excited signal; blue, 405-nm-excited signal) either following tail lift (left, tail lift) or without simulation (right: no stim.). The regions covering s.d. values of the traces are shaded in light colors. Tail lift was applied during the periods shaded pink. **g**, Summary of AUC values during stimulation (*n* = 18 trials from three mice). Statistics used were one-way ANOVA (*F*_3,68_ = 2.74, *P* = 7.5 × 10^−14^) with Bonferroni post hoc test (*P* = 5.0 × 10^−5^, 470 versus 405 in tail lift, *P* = 1 in 470 versus 405 in no stim.). **h**, Summary of times to peak for tail lift-induced OT responses (*n* = 18 trials from three mice). Scale bar, 200 µm (**e**). Graphs represent the mean ± s.e.m. (**c** and **g**) and mean ± s.d. (**b**, **d**, **f** and **h**). ***P < 0.001, *P < 0.05, NS (**c** and **g**). Source data

Taking advantage of the rapid kinetics of MTRIA_OT_, we also tested whether the sensor can detect faster phenomena. Interaction with a conspecific animal results in prompt activation of OT neurons^34^; therefore, we examined social interaction-induced MTRIA_OT_ responses in the AON using fiber photometry (Fig. 4a). Following an encounter with a conspecific mouse enclosed in a wire cage, the fluorescence signal of MTRIA_OT_ started to gradually increase, which was characterized by a rise time constant of ~1 min. We also verified that there was no apparent increase in the signal when stimulated with a toy mouse (Fig. 4b–d). These results indicate that MTRIA_OT_ can be used to detect OT responses with a minute-order time constant.

We next explored faster OT responses. OT levels are thought to increase in response to a wide variety of stressful stimuli^35^. A major target of stress-induced OT signals is the central nucleus of the amygdala^30,35^; therefore, we virally expressed MTRIA_OT_ around this region and measured OT responses after a tail lift as a rapid stress stimulus (Fig. 4e). After lifting the tail of a mouse, we observed a rapid rise in MTRIA_OT_ signal. Such robust responses were absent in simultaneously recorded 405-nm-excited reference signals, as well as 470-nm-excited and 405-nm-excited signals recorded without stimulation, indicating artifacts are negligible (Fig. 4f,g). The mean time to reach peak signal value was approximately 6 s (Fig. 4h), demonstrating the utility of MTRIA_OT_ to detect OT responses that rapidly appear within a few seconds. Taken together, the above results show the potential of MTRIA_OT_ for analyzing OT responses during various timescales.

### Examination of potential factors affecting brain oxytocin dynamics

**Fig. 5:**
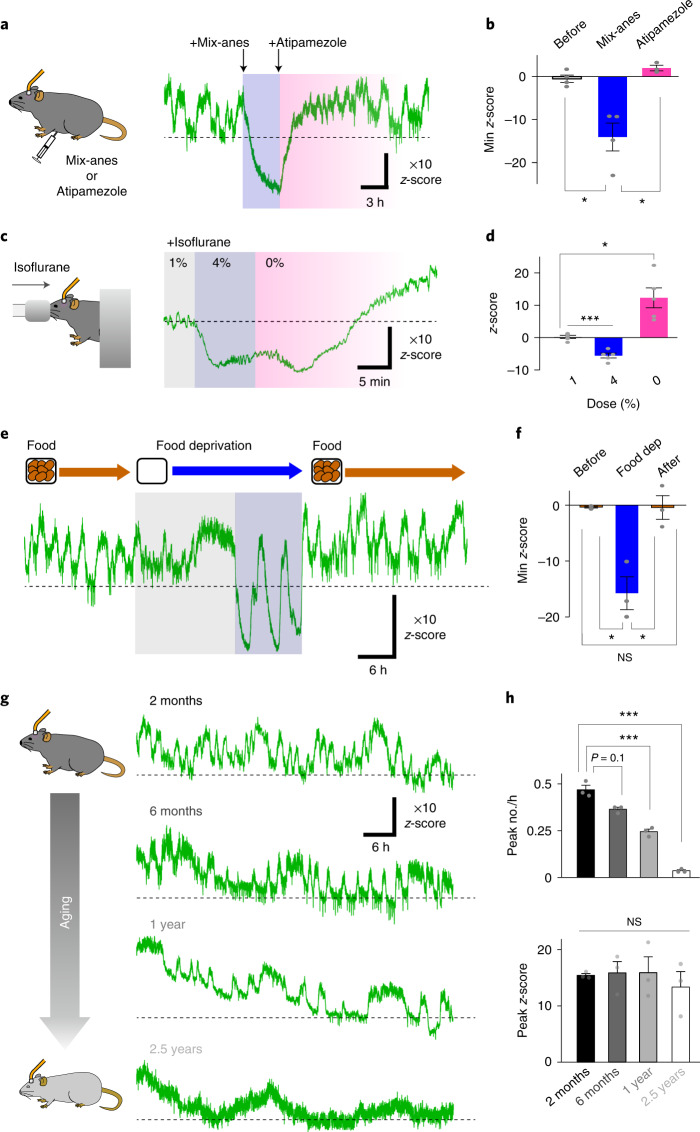
Alterations in brain oxytocin levels caused by anesthesia, food deprivation and aging. **a**, Schematic illustrating the recording and representative trace of MTRIA_OT_ activity showing the impact of mix-anes on OT oscillation. The background of the trace is shaded to indicate the period of anesthesia after mix-anes administration (dark blue) and period after release by the antagonist, atipamezole (pink). **b**, Summary of minimum *z*-score values before mix-anes administration, after administration of mix-anes and following subsequent administration of atipamezole (*n* = 4 mice in before and mix-anes, *n* = 3 mice in atipamezole). **c**, Schematic of the recording and representative trace of MTRIA_OT_ activity showing the impact of isoflurane on OT signal. The background of the trace is shaded to indicate the period of 1% isoflurane administration (gray), of 4% isoflurane administration (dark blue), and after release from the anesthesia (0%, pink). **d**, Summary of the tail values of *z*-scores during the above three states (*n* = 5 mice). **e**, Representative trace of MTRIA_OT_ activity showing the impact of food deprivation on OT oscillation. The background of the trace is shaded to indicate the period of food deprivation. The period of OT turbulence is colored dark blue and the peaks of undershot signal are indicated by arrowheads. **f**, Summary of minimum *z*-score values before, during and after food deprivation (*n* = 3 mice). **g**, Representative traces of MTRIA_OT_ fluorescence signals in mice at the indicated age (2 months, 6 months, 1 year or 2.5 years). **h**, Summary of the peak number every hour (top) and peak *z*-score (bottom; *n* = 3 mice). Graphs represent the mean ± s.e.m. (**b**, **d**, **f** and **h**). ***P < 0.001, *P < 0.05 and NS unless otherwise stated (**b**, **d**, **f** and **h**). Statistics (**b**, **d**, **f** and **h**) are summarized in Supplementary Note 3. Source data

**Extended Data Fig. 7:**
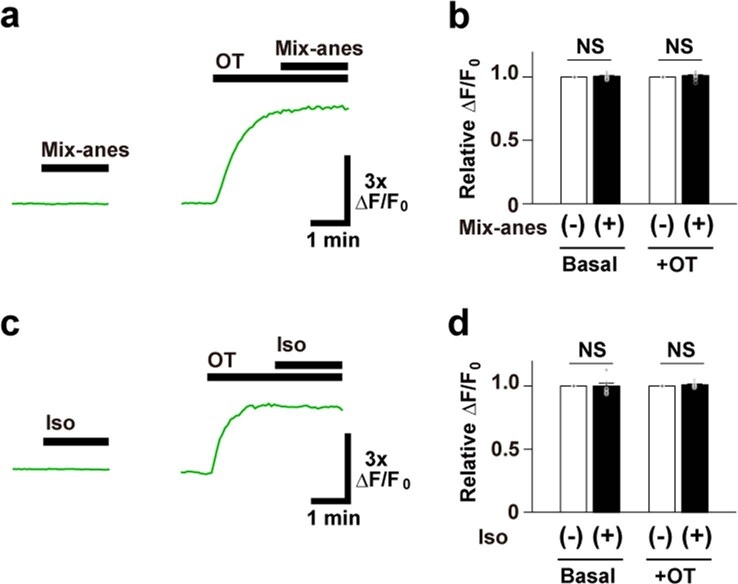
Effects of anesthetic drugs on MTRIA_OT_ fluorescence level in HEK293T cells. **a**, Representative traces (left: basal fluorescence level, right: OT-induced response) showing the dependence of MTRIA_OT_ fluorescence intensity on mix-anes (0.11 mg/mL dexmedetomidine hydrochloride, 0.6 mg/mL midazolam, and 0.75 mg/mL butorphanol tartrate). **b**, Summary of Δ*F*/*F*_0_ responses by the value just before mix-anes application (*n* = 10 cells). Statistics: paired two-tailed *t*-test (*P* = 0.48: Basal, *P* = 0.24: +OT). **c**, Representative traces (basal fluorescence level: left, OT-induced response: right) showing the dependence of MTRIA_OT_ fluorescence intensity on isoflurane (1:100 dilution). **d**, Summary of Δ*F*/*F*_0_ responses by the value just before isoflurane application (*n* = 10 cells). Statistics: paired two-tailed *t*-test (*P* = 0.97: Basal, *P* = 0.18: +OT). Graphs represent mean ± SEM (**b**, **d**). NS: not significant (**b**, **d**). Source data

We next explored potential factors affecting patterns of OT dynamics in the brain. General anesthetics exert substantial effects on neurotransmission^36,37^; therefore, we evaluated the impact of two anesthetics on OT levels in the AON using fiber photometry recording. First, we examined the effect of a mixture of dexmedetomidine, butorphanol and midazolam (mix-anes). When mix-anes was intraperitoneally administered, the fluorescence signal of MTRIA_OT_ fell to a level below the baseline (Fig. 5a,b). The undershot signal of MTRIA_OT_ was reversed to a level comparable to baseline after administration of atipamezole, a drug that has an antagonistic effect on mix-anes (Fig. 5a,b). We also examined the effect of isoflurane, an inhalation anesthetic, on MTRIA_OT_ responses by sequentially changing the dose from 1% to 4% to 0%. Consistent with the above result, the level of MTRIA_OT_ fluorescence became lower as the dose of the anesthetic increased (Fig. 5c,d). We confirmed that mix-anes and isoflurane did not affect basal or OT-evoked fluorescent signals in HEK293T cells (Extended Data Fig. 7), excluding the possibility of direct suppressive effects of these anesthetics on the fluorescence signal of the sensor. Together, these results demonstrate the suppressive effect of anesthesia on brain OT levels.

Feeding is associated with OT release^38^; therefore, we examined the impact of fasting stress on brain OT dynamics. After about half a day of food deprivation, patterns of OT oscillation gradually became disturbed and the oscillatory signals undershot to a level below the initial baseline (Fig. 5e,f). We named this phenomenon ‘OT turbulence’ (Fig. 5e). After refeeding, OT turbulence halted and normal OT oscillation quickly recovered (Fig. 5e,f). Considering the role of OT as an appetite suppressant^39^, OT turbulence may manage hunger stress during starvation.

**Extended Data Fig. 8:**
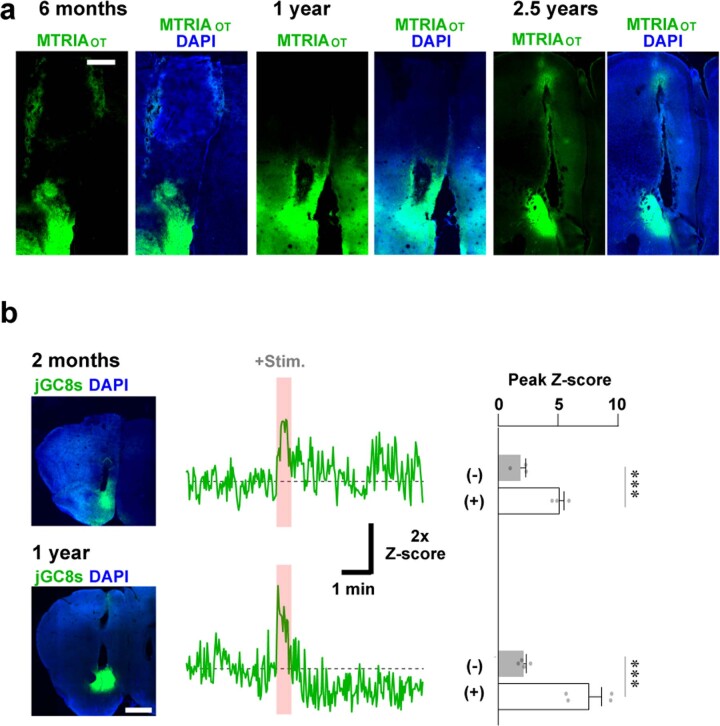
Verification of sensor expression and viability of brain tissue following surgery in aged mice. **a**, Histology showing transduction of MTRIA_OT_ in the AON of mice at 6 months, 1 year, and 2.5 years of age (green: MTRIA_OT_, blue: DAPI). **b**, Data on fiber photometry recordings of odor-induced Ca^2+^ responses in the AON using mice aged either 2 months (top) or 1 year (bottom). Histology showing jGCaMP8s expression (left), representative traces of the recordings (middle), and summary of peak z-scores of the jGCaMP8s signals before (-) and during (+) odor simulation are summarized (*n* = 3 mice for 2 months and *n* = 4 mice for 1 year), respectively. As an odor stimulant, a small piece of a filter paper soaked with limonene was displayed for 30 s during the period shown in pink in the middle panel. Statistics: unpaired two-tailed *t*-test (*P* = 0.006: 2 months, *P* = 0.002: 1 year). Graphs represent mean ± SEM (**b**). ***P < 0.01 (**b**). Scale bars, 500 µm (**a**) and 1 mm (**b**). Source data

Aging is associated with a decline in the neuroendocrine system^3,40^; therefore, we next analyzed differences in AON OT dynamics in mice at varying ages (~2 months, ~6 months, ~1 year and ~2.5 years). We verified that AAV-mediated transduction of the sensor was successful in old animals (Extended Data Fig. 8a). Fiber photometry-mediated fluorescence measurements revealed the occurrence of oscillatory OT responses in all groups, but the frequency of OT transients became slower in older animals, although there were no detectable changes in amplitude (Fig. 5g,h). It may be possible that the reduced frequency of OT oscillation results from the vulnerability of tissue in older animals to the surgery required for fiber photometry recording. However, odor-induced Ca^2+^ responses in the AON were robust even in 1-year-old mice (Extended Data Fig. 8b), supporting the validity of our fiber photometry recordings in old mice. These results indicate that an altered frequency of OT oscillation may underlie aging-associated decline of brain function.

### Developing other G-protein-coupled receptor-based sensors using MTRIA

**Fig. 6:**
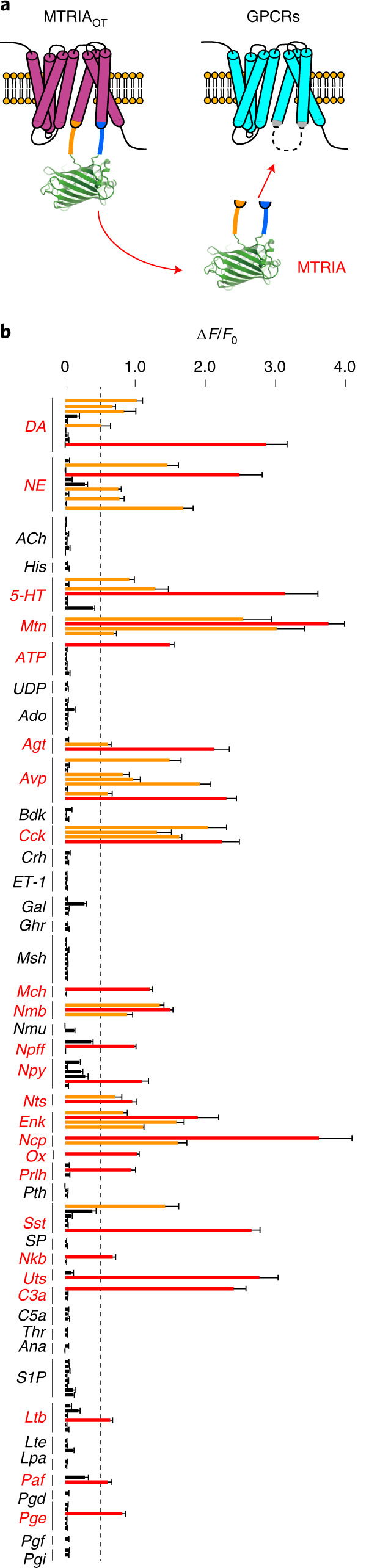
Development of various G-protein-coupled receptor-based sensors by conjugation with MTRIA. **a**, Schematic illustrating the development of MTRIA sensors. **b**, Δ*F*/*F*_0_ values are given as the mean ± s.e.m. (*n* = 5 cells for each group). Sensors with Δ*F*/*F*_0_ > 0.5 are colored either red or orange. Sensors with the best performance among receptors sharing the same ligand are colored red. Source data

**Extended Data Fig. 9:**
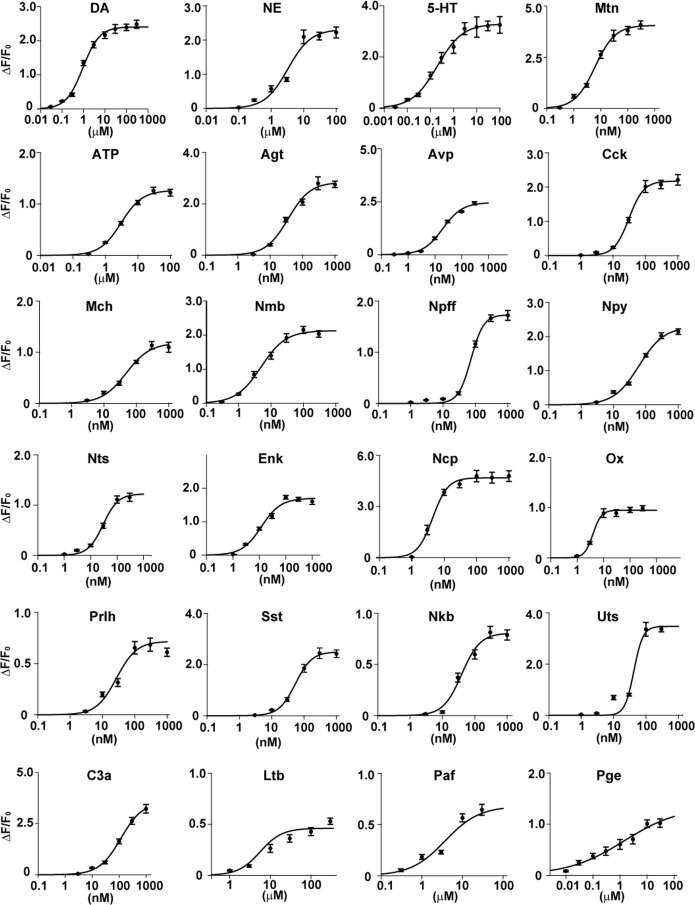
Dose-response curves of MTRIA sensors. Dose-response curves of the fluorescent sensors for 24 ligands that were examined in HEK293T cells. Data are shown as mean ± SEM (*n* = 10 cells for each data point). Ligands were as follows: dopamine (DA), norepinephrine (NE), serotonin (5-HT), melatonin (Mtn), adenosine 5′-triphosphate (ATP), angiotensin (Agt), arginine-vasopressin (Avp), cholecystokinin (Cck), melanin-concentrating hormone (Mch), neuromedin B (Nmb), neuropeptide FF (Npff), neuropeptide Y (Npy), neurotensin (Nts), enkephalin (Enk), nociception (Ncp), orexin (Ox), prolactin-releasing hormone (Prlh), somatostatin (Sst), neurokinin B (Nkb), urotensin (Uts), complement component C3a (C3a), leukotriene B (Ltb), platelet-activating factor (Paf), and prostaglandin E (Pge). Source data

MTRIA was able to successfully produce a sensitive optical readout of OTR activation; therefore, we examined whether MTRIA was also able to detect ligand-binding-induced conformational changes of other GPCRs (Fig. 6a). We cloned 184 receptors for 46 ligands derived from human, mouse, zebrafish or medaka and conjugated MTRIA to a region ranging from position 5.62 of TM5 to position 6.36 of TM6 in the receptors (Extended Data Fig. 2a). To our surprise, almost 30% of the engineered sensors (54 of 184 proteins) showed a marked fluorescence increase (>50% Δ*F*/*F*_0_) upon stimulation with high concentrations of their specific ligand (Fig. 6b). In Extended Data Fig. 9 and Supplementary Table 1, we named the 24 sensors that showed the largest fluorescence response among the sensors sharing the same ligand MTRIA sensors (for example, a sensor for dopamine was called MTRIA_DA_). We hope that this approach, the MTRIA system, will help accelerate the engineering of various GPCR-based sensors.

## Discussion

In this study, we described the development and application of a fluorescent sensor for OT, MTRIA_OT_. MTRIA_OT_-mediated in vivo fluorescence recording can report artificially evoked OT responses as well as endogenously controlled OT signals with a fast temporal resolution.

Although the impacts of exogenous OT administration on mood and emotions are expected to lead to treatments for psychiatric disorders, direct evidence for an increase in brain OT levels following peripheral OT administration remains limited. However, neither intranasal nor intraperitoneal administration of a relatively large amount had a substantial effect on OT levels in the AON. Further validation is needed to clarify whether this is the case for other brain regions.

Our in vivo measurements using MTRIA_OT_ revealed OT oscillation, ultradian OT rhythms with intervals of approximately 2 h in the brains of adult mice. These type of OT dynamics have not been reported using conventional approaches, such as microdialysis, presumably because of the limited sampling rate and sensitivity. Future efforts are needed to clarify the regulatory mechanism and physiological significance of OT oscillation. Given that hormones associated with appetite and metabolism can stimulate OT release^38,41^, the rhythmic activities of brain OT signals may be autonomically controlled via interoceptive signals associated with feeding behaviors. Our observations of disturbance in the patterns of OT oscillation by food deprivation and aging support this idea.

An issue to be addressed in the future is the quantitative measurement of OT concentrations in the living brain. This will help to directly compare data acquired from different measurement sites, in different animals, or from different experimental setups. Development of next-generation OT sensors dependent on changes in quantitative fluorescence parameters (for example, fluorescence lifetime and efficiency of Förster resonance energy transfer)^42,43^ will offer an opportunity for real-time monitoring of absolute OT quantities in the living brain.

Overall, our MTRIA_OT_-mediated measurements provide information about OT dynamics in the brain. Moreover, our analyses indicate that patterns of OT dynamics in the brain can be affected by anesthesia, food deprivation and aging. Therefore, it is important to carefully consider the experimental conditions and state of participants when interpreting OT measurement data in the brain. Indeed, context-dependent and participant-dependent inconsistencies regarding the effects of OT in human clinical trials^44^ may be related to such parameters. The use of MTRIA_OT_ will allow us to extend our knowledge of brain OT dynamics and its association with complex behaviors.

## Methods

### DNA constructs

DNA encoding GPCRs, a red fluorescent Ca^2+^ sensor jRGECO1a^45^ and PM-targeted mScarlet^46^ (mSca_mem_) were amplified by PCR and then subcloned into the pCIS vector, a CAG promoter-driven expression vector^47^. Template DNA for GPCRs was prepared using the following four approaches. First, cDNA libraries of a mouse (*Mus musculus*) brain, zebrafish (*Danio rerio*) brain and medaka (*Oryzias latipes*) brain were prepared using an RNeasy Kit (Qiagen) and QuantiTect Reverse Transcription Kit (Qiagen). Second, genomic DNA of human (*Homo sapiens*) and mouse were isolated from HEK293T cells and a mouse brain, respectively. Third, gene fragments of OTRs derived from chicken (*Gallus gallus*), snake (*Notechis scutatus*) and frog (*Xenopus laevis*) were synthesized by Integrated DNA Technologies. Finally, for cloning of human dopamine receptor *DRD1*, human muscarinic receptor *CHRM3* and human adrenergic receptor *ADRA2A*, plasmids encoding dLight1.1, GACh2.0 and GRAB_NE_1m were used as templates, respectively^14–16^. GPCRs used in Fig. 6 and the primer pairs for their cloning are listed in Supplementary Table 2. To assess localization of OTRs in HEK293T cells, a human influenza hemagglutinin (HA)-tag was added to the N-terminal regions of OTRs using PCR. For the construction of green fluorescent transmitter sensors, cpGFPs derived from GCaMP6s were inserted into the IL3 regions of GPCRs using either overlap extension PCR^48^ or the Gibson assembly protocol^49^. For mutant sensor screening, site-directed mutagenesis was performed using primers containing mutated nucleotide(s). In particular, randomized site-directed mutagenesis was achieved using primers containing randomized codons (NNN). For the construction of plasmid DNA in the β-arrestin recruitment assay, fragments of NanoLuc—SmBit and LgBit—were amplified from pNL1.1 (Promega). Subsequently, they were fused with the C terminus of the OTR and the N terminus of rat β-arrestin^50^, followed by cloning into the pCIS vector. For the construction of transfer plasmid DNA for AAVs, MTRIA_OT_, MTRIA_OT_-mut and jGCaMP8s were cloned into the pAAV Syn woodchuck hepatitis virus posttranscriptional regulatory element (WPRE) vector, a plasmid driven by a human synapsin promoter. In addition, mSca, TeLC-P2A-mSca and ChRmine–mSca were cloned into the pAAV-OTp-WPRE vector, a plasmid driven by the mouse OT promoter.

### Cell culture

HEK293T cells were cultured in DMEM (FUJIFILM Wako) supplemented with 10% fetal bovine serum (Thermo Fisher Scientific), 100 U ml^−1^ penicillin and 100 µg ml^−1^ streptomycin at 37 °C under a humidified atmosphere of 5% CO_2_ and 95% air. For fluorescence microscopy observations, cells were transfected with 1 µg of plasmid using 2 µg of a polyethylenimine-based transfection reagent (PEI MAX; Polysciences) and seeded onto either glass-bottom dishes or glass-bottom chambers (Matsunami) coated with collagen (Nitta Gelatin). After a 3-h incubation, the medium was changed, and cells were further cultured for 24–36 h before imaging experiments. Rat primary cultured neurons were prepared from the hippocampus of embryonic day 19–20 Sprague Dawley rats (CLEA Japan). Briefly, the dissected hippocampus was digested in Hanks’ Balanced Salt Solution (HBSS) containing 1 unit per ml of papain (Sigma), 0.45 mg ml^−1^ l-cysteine (FUJIFILM Wako) and 0.1 mg ml^−1^ DNase I (FUJIFILM Wako) at 37 °C for 30 min and then passed through a 70-μm nylon mesh. Isolated cells were resuspended in Neurobasal Medium (Thermo Fisher Scientific) containing NeuroBrew 21 (1:50 dilution; Miltenyi), GlutaMax (1:100 dilution; Thermo Fisher Scientific), 100 U ml^−1^ penicillin and 100 µg ml^−1^ streptomycin and were seeded on glass-bottom dishes coated with laminin (FUJIFILM Wako) and poly-l-lysine (FUJIFILM Wako). After 3 d in vitro, fresh medium, an AAV for MTRIA_OT_ transduction and cytosine-1-β-d-arabinofuranoside (final 5 µM; FUJIFILM Wako) were applied. Then, half the amount of medium was replaced every 7 d until experiments were conducted at 12–19 d in vitro.

### Adeno-associated virus production

HEK293T cells were simultaneously transfected with pHelper, XR8 and a transfer plasmid using PEI MAX transfection reagent, except for production of a viral vector for jGCaMP8s where XR9 was used instead of XR8. After overnight incubation, the medium was changed. Supernatant was collected at 48-h and 96-h time points, after medium exchange. Viral particles were purified using a polyethylene glycol-mediated precipitation method^51^. Purified viral particles were then concentrated using an ultrafiltration membrane unit (Amicon Ultra; EMD Millipore). Virus titers were determined by quantitative PCR using a pair of primers for the WPRE sequence (forward, actgtgtttgctgacgcaac; reverse, agcgaaagtcccggaaag). Viral vectors with titers > 10^12^ gc per ml were used for fiber photometry measurements.

### Immunostaining of transfected cells

For immunostaining analysis of the cellular localization of OTRs, HEK293T cells co-transfected with an HA-tagged OTR and mSca_mem_ (10:1 ratio) were fixed in PBS (FUJIFILM Wako) containing 4% (wt/vol) paraformaldehyde for 10 min at room temperature. Next, cells were permeabilized and blocked for 30 min at room temperature in blocking solution (PBS containing 0.2% TritonX-100 and 5% normal goat serum (FUJIFILM Wako)) and then incubated with an anti-HA antibody (rabbit; MBL; 1:2,000 dilution) for 30 min at room temperature. After washing with PBS containing 0.1% TritonX-100 (PBST), samples were incubated with Alexa 488-conjugated anti-rabbit IgG antibody (goat; Jackson ImmunoResearch) for 30 min at room temperature. After three washes with PBST, stained cells in PBS were analyzed by microscopy.

### Sensor screening and evaluation by fluorescence microscopy

Fluorescence images in Figs. 1 and 6 and Extended Data Figs. 1, 3a–e, 5 and 9 were obtained using Dragonfly 301, a spinning-disk confocal microscope system (Andor Technology) equipped with a Nikon Eclipse Ti2, lasers (405, 488, 561 and 637 nm), emission filters (450 ± 25 nm for the 405-nm laser, 525 ± 25 nm for the 488-nm laser, 600 ± 25 nm for the 561-nm laser and 700 ± 37.5 nm for the 637-nm laser) and an electron multiplication charge-coupled device camera (iXon Life 888; Andor Technology). The system was controlled by Fusion software (Andor). Either a dry objective lens (CFI PLAN APO ×20, NA 0.75; Nikon) or a water-immersion lens (CFI Plan Apo IR 60XC WI, NA 1.27; Nikon) was used.

The fluorescence images in Fig. 2g and Extended Data Figs. 3f,g, 4 and 6–8 were obtained using SpinSR10, a spinning-disk confocal microscope system (Olympus) equipped with lasers (at 405, 488, 561 and 637 nm; Coherent), a scanner unit (CSU-W1 SoRa; Yokogawa) and a complementary metal oxide semiconductor camera (Orca Flash 4.0, Hamamatsu Photonics). The system was controlled by cellSens software (Olympus). An objective lens (UPlanSApo ×10, NA 0.40; UPlanSApo ×20, NA 0.75; or UPlanSApo ×30, NA 1.05 Sil; Olympus) was used. For live-imaging experiments, data were acquired either every 100 ms (for kinetic analysis in Extended Data Figs. 3f and 4e,f) or every 5 s (for other experiments). In these experiments, laser power was 200 µW or less at the front aperture of the objective lens, and no apparent photobleaching was observed with these conditions. Therefore, we did not conduct photobleaching correction. To analyze the cellular localization of fluorescent sensors, *z*-stack images were acquired at 0.5-µm steps. For the screening of fluorescent sensors, transfected cells seeded onto glass-bottomed chamber slides were soaked in 200 µl of artificial extracellular solution (ECS; 150 mM NaCl, 4 mM KCl, 2 mM CaCl_2_, 1 mM MgCl_2_, 5 mM HEPES and 5.6 mM glucose; pH 7.4). Cells were then stimulated with 200 µl of drug-containing ECS, in which the drug concentration was twice the final concentration, administered through a pipette. To analyze the ligand dose–response curves of fluorescent sensors, time constants of fluorescent responses, intracellular Ca^2+^ responses, pH sensitivity and long-term signal stability, transfected cells were seeded onto a collagen-coated glass-bottomed dish and continuously perfused with ECS. Cells were then rapidly stimulated with drug-containing solution using a custom-made perfusion system equipped with solenoid valves. For the measurement of kinetic time constants, the tip of the perfusion system was placed as close to the imaged cells as possible. Data were processed using Fiji software^52^ and Excel (Microsoft), and fitting of fluorescence traces was conducted using Python modules. After conducting background subtraction, fluorescence signal (*F*) on a single cell was obtained. Signals were normalized to Δ*F/F*_0_ defined by (*F* − *F*_0_)/*F*_0_ where *F*_0_ is the fluorescence value just before stimulation. Fluorescence traces were not smoothed. Values of Δ*F/F*_0_ in Figs. 1e,h and 6b, and Extended Data Figs. 3b,g, 4c,d, 7b,d and 9 were quantified as the tail values at the end of drug stimulation. Values of peak Δ*F/F*_0_ in Fig. 1g and Extended Data Fig. 3c were quantified as the maximum values during drug stimulation. Parameters of dose–response curves (*F*_max_ and EC_50_ in Fig. 1h, Extended Data Figs. 3b and 4d and Supplementary Table 1) were obtained by fitting the data to the Hill equation. Time constants (τ_on_ and τ_off_) in Extended Data Figs. 3f and 4e,f were calculated by fitting rise and decay phases with single-exponential functions, respectively.

### β-arrestin recruitment assay

The recruitment of β-arrestin to meOTR and MTRIA_OT_ was assessed using a split-luciferase complementation assay. HEK293T cells that were seeded onto an opaque 96-well plastic plate were co-transfected with LgBit-arrestin and either meOTR-SmBit or MTRIA_OT_-SmBit using PEI MAX reagent. Twenty-four hours after transfection, cells were washed with HBSS and stimulated with HBSS containing 100 nM OT. After the addition of a luciferase substrate (ONE-Glo Luciferase Assay System, Promega), luminescence was measured using a plate reader (Infinite 200 Pro F Plex; Tecan).

### Reagents

OT, angiotensin (Agt), [Arg^8^]-vasopressin (Avp), bradykinin (Bdk), cholecystokinin (Cck), corticotropin-releasing hormone (Crh), endothelin-1 (ET-1), galanin (Gal), ghrelin (Ghr), α-melanocyte stimulating hormone (Msh), melanin-concentrating hormone (Mch), neuromedin B (Nmb), neuromedin U (NmU), neuropeptide Y (Npy), neurotensin (Nts), met-enkephalin (Enk), nociceptin (Ncp), orexin B (Ox), prolactin-releasing hormone (Prlh), parathormone (Pth), somatostatin (Sst), substance P (SP), neurokinin B (Nkb) and urotensin (Uts) were purchased from Peptide Institute. L-368,899 hydrochloride, neuropeptide FF (NpFF), sphingosine-1-phosphate (S1P), uridine 5′-diphosphate disodium salt (UDP) and anandamide (Ana) were purchased from Tocris Bioscience. Isotocin, C3a anaphylatoxin (C3a), and C5a anaphylatoxin (C5a) were purchased from Bachem. Vasotocin and thrombin receptor agonist (Thr) were purchased from Anygen, and inotocin was purchased from Phoenix Pharmaceuticals. Histamine disodium salt (His) and melatonin (Mtn) were purchased from FUJIFILM Wako. Leukotriene B4 (Ltb), leukotriene E4 (Lte), 1-oleoyl lysophosphatidic acid (LPA), platelet-activating factor C-16 (PAF) and prostaglandin F2a (Pgf) were purchased from Cayman Chemicals. Prostaglandin D2 (Pgd), prostaglandin E2 (Pge), prostaglandin I2 sodium salt (Pgi), 5-hydroxytryptamine hydrochloride (5-HT), dopamine hydrochloride (DA), acetylcholine chloride (ACh) and adenosine 5′-triphosphate disodium salt (ATP) were purchased from Nacalai. Epinephrine was purchased from Daiichi-Sankyo and norepinephrine (NE) was purchased from Alfresa Pharma Corporation. Nematocin was synthesized by Cosmo Bio. The final concentrations of applied ligands in Fig. 6b were as follows; 100 µM: DA, NE, ACh, His, 5-HT, ATP, UDP and Ado; 1 µM: Enk, C3a, Ana, LPA, PAF, Pgd, Pge, Pgf and Pgi; and 100 nM: Mtn, Agt, Avp, Bdk, Cck, Crh, ET-1, Gal, Ghr, Msh, Mch, Nmb, NmU, NpFF, Npy, Nts, Ncp, Ox, Prlh, Pth, Sst, SP, Nkb, Uts, C5a, Thr, S1P, Ltb and Lte.

### Animal surgery

Animal experiments were reviewed and approved by Institutional Animal Use and Care Committees of Kanazawa University and Osaka University. All animal procedures were conducted in accordance with the guidelines of Kanazawa University and Osaka University. C57BL/6J or C57BL/6N female mice at 6–8 weeks, 6 months, 1 year or 2.5 years of age were purchased from CLEA Japan. Mice were kept in cages at 23 ± 1.5 °C with 45% ± 15% humidity under a regular 12-h dark/light cycle with ad libitum access to food and water. Stereotaxic surgery was performed under anesthesia with isoflurane (FUJIFILM Wako). The depth of anesthesia was assessed using the tail pinch method. Body temperature was maintained using a heating pad. One microliter of AAV suspension within a glass micropipette was injected into the corresponding site(s); left medial AON (2.2 mm anteroposterior (AP) and 0.3 mm mediolateral (ML) from bregma, and −4.0 mm dorsoventral (DV) from the skull surface), left and right PVN (−0.8 mm AP, ±0.3 mm ML and −4.8 mm DV), and right central nucleus of the amygdala (−1.3 mm AP, 2.5 mm ML and −4.5 mm DV). After the virus injection, a fiber-optic cannula with a 400-µm core diameter and 0.39 NA (CFMC14L05; Thorlabs) was implanted ~0.2 mm above the injection site. For optogenetic experiments, a cannula was unilaterally implanted above the injection site of the right PVN. For intracerebroventricular injection experiments, a stainless-steel cannula fabricated from a 22-gauge needle was additionally inserted into the right lateral ventricle (−0.7 mm AP, 1.5 mm ML and −2.5 mm DV). To fix and protect the implanted cannula(s), dental cement (Ketac Cem Easymix; 3 M) and silicone rubber (Body Double; Smooth-On) were used. Carprofen (5 mg per kg body weight; intraperitoneal), a nonsteroidal anti-inflammatory drug, and buprenorphine (0.1 mg per kg body weight, intraperitoneal), an opioid analgesic, were administered after the surgery. At 2 weeks or more following AAV injection, mice were used for fiber photometry experiments. On the measurement day, the cannula for fiber photometry recording was coupled to a patch cable (M79L01; Thorlabs) via an interconnector (ADAF1 or ADAF2; Thorlabs). For optogenetic experiments, the cannula for optogenetic stimulation was additionally connected to a red laser (OBIS LX, 647 nm, 100 mW; Coherent) through a patch cable (MAF3L1; Thorlabs) equipped with an interconnector (ADAF1).

### Fiber photometry measurements

The fiber photometry setup (Extended Data Fig. 5a) was constructed as previously described, with a minor modification^53^. Excitation light from a light-emitting diode (LED) was directed into a patch cable (M79L01) through an objective lens (CFI Plan Fluor ×20 lens; Nikon), and the emission light was projected onto the sensor of a scientific complementary metal oxide semiconductor camera (Zyla 4.2 P; Andor Technology) after passing through a dichroic mirror (Di01-R405/488/561/635-25×36; Semrock) and emission filter (YIF-BA510-550S; SIGMA KOKI). To acquire an OT-dependent signal and isosbestic signal, two different LED light sources—a 470-nm light (light from M470F3 filtered with FB470-10, 4 µW at the fiber tip; Thorlabs) and 405-nm light (light from M405FP1 filtered with FB410-10, 4 µW at the fiber tip; Thorlabs)—were alternatively switched. Data were acquired at 2-s intervals with an LED light exposure of 0.3 s except for measurements of fast responses (Fig. 2f–j and 4e–h), where data were acquired at 0.5-s intervals with an LED light exposure of 0.08 s. In these experimental conditions, no apparent photobleaching was observed. Therefore, we did not conduct photobleaching correction. Device control and data acquisition were conducted using a custom-made LabVIEW program (National Instruments). Experiments were conducted within a cage (270-mm width, 440-mm length and 187-mm height), and the environment around the cage was captured every 1 s using an overhead camera (BFS-U3-I3Y3-C; FLIR Systems). To visualize the behaviors of mice and timing of simulations in the dark environment, light from an infrared LED array (AE-LED56V2; Akizuki) was used. Unless stated otherwise, mice were kept in freely behaving conditions and fed standard pellet and water ad libitum during measurements. For recording of social stimulation-induced OT responses (Fig. 4a–d), a cylindrical wire cage (10-cm diameter and 10-cm height) containing either a mouse or a toy was exposed to a mouse at the indicated times. Similarly, for recording acute stress-induced OT responses (Fig. 4e–h), the tail of a mouse was lifted for 10 s at the indicated times. To assess OT responses upon exogenous OT administration (Fig. 2a–e) and the impact of mix-anes (Fig. 5a,b), mice were anesthetized by intraperitoneal administration of a mixture of 0.375 mg per kg body weight dexmedetomidine hydrochloride, 2 mg per kg body weight midazolam and 2.5 mg per kg body weight butorphanol tartrate. For recovery from the above anesthesia, 0.75 mg per kg body weight atipamezole hydrochloride was administrated intraperitoneally. Before starting optogenetic experiments (Fig. 2f–j) and assessment of the impact of isoflurane (Fig. 5c,d), mice were placed on a heat pad and maintained with 1% isoflurane for at least for 20 min to stabilize the fluorescence signal of MTRIA_OT_. Data were processed using Fiji software and Excel (Microsoft), and kinetic parameters were calculated using Python modules. After conducting background subtraction, the fluorescence signal (*F*) was normalized to *z*-scores defined by (*F* − *F*_mean_)/*F*_sd_, where *F*_mean_ and *F*_sd_ are the mean and standard deviation of resting fluorescence signals at baseline, respectively. Resting fluorescence signals were taken for the indicated period as follows: 5 s (Fig. 4f–h), 10 s (Fig. 2h–j), 1 min (Fig. 4b–d), 5 min (Fig. 5c,d and Extended Data Fig. 8b) and 10 min (Figs. 2b–e, 3 and 5e–h). Data were not smoothed except for the fluorescence traces in optogenetic experiments (Fig. 2h–j). For analysis of fluorescence signals in optogenetic experiments, data were filtered using a moving average filter with a kernel (1/5, 1/5, 1/5, 1/5, 1/5) to improve the quality of single-exponential fitting. Time constants (τ_on_ and τ_off_ in Fig. 2j and Rise τ in Fig. 3d) were calculated by fitting either the rise phase or the decay phase with a single-exponential function. Time to peak in Fig. 4h was obtained as a period from the beginning of tail lift to the time point of maximal *z*-score. AUC values in Figs. 2i and 4c,g were calculated by integrating the values of *z*-score for 30 s, and 5 min and 10 s, respectively. *Z*-scores in Figs. 2c and 5d were quantified as the tail values at the end of the indicated stimulation. Peak *z*-scores in Fig. 2e and Extended Data Fig. 8b were quantified as the peak values following stimulation. The remaining peak *z*-scores (Figs. 3c,h and 5h) and minimum *z*-scores (Fig. 5a,f) were obtained as 10-min average values around the peak and the minimum of transient OT responses, respectively. The numbers of peaks in Figs. 3g and 5h were calculated as an occurrence of transient signal with an amplitude exceeding a *z*-score of 3.

### Immunohistochemistry

Immunohistochemical experiments were performed to confirm sensor expression and cannula insertion sites after fiber photometry measurements and to assess the specificity of OTp-mediated gene transduction. The brains of anesthetized mice were rapidly removed after decapitation, immersed in PBS containing 4% (wt/vol) paraformaldehyde overnight at 4 °C, and then incubated in PBS containing 20% (wt/vol) sucrose overnight at 4 °C. After embedding in Optimal Cutting Temperature Compound (Sakura Finetek), coronal sections of brains were cut at either a 50-µm (for anti-GFP antibody staining) or a 30-µm (for anti-OT antibody staining) thickness using a cryostat (CM1950; Leica Microsystems). For immunostaining, sections were permeabilized in blocking solution for 30 min at room temperature and then incubated with either anti-GFP antibody (rabbit; MBL; 1:1000 dilution) or anti-OT antibody (rabbit; Abcam; 1:200 dilution) overnight at 4 °C. After three washes with PBST, sections were incubated with Alexa 488-conjugated anti-rabbit IgG antibody (goat; Jackson ImmunoResearch; 1:2,000 dilution) for 40 min at room temperature. After three washes with PBST and three washes with PBS, samples were mounted in polyvinyl alcohol-based mounting medium containing 1 µg ml^−1^ DAPI (FUJIFILM Wako) and 2.5% (wt/vol) 1,4-diazabicyclo[2.2.2]octane (Sigma) as an anti-fade reagent. Stained sections were then imaged using a spinning-disk confocal microscope system (either Dragonfly 301 or SpinSR10). Mice with misplaced cannula were excluded from analysis.

### Statistics and reproducibility

All summary data are expressed as either the mean ± s.e.m. or mean ± s.d. For comparisons of two groups, we used two-tailed Student’s *t*-tests. One-way ANOVA was performed when more than two groups were compared, followed by Tukey–Kramer, Bonferroni’s or Dunnett’s post hoc test. Throughout the study, *P* < 0.05 was considered statistically significant. Data distribution was assumed to be normal, and variance was similar between groups that were statistically compared. No statistical methods were used to predetermine sample sizes, but our sample sizes were similar to those generally used in the field^13–17^. Regarding the representative data in Figs. 1e, 2g and 4e and Extended Data Figs. 4a,b, 5b, 6 and 8a, we repeated the experiments two or more times and obtained similar results.

### Reporting summary

Further information on research design is available in the Nature Research Reporting Summary linked to this article.

## Online content

Any methods, additional references, Nature Research reporting summaries, source data, extended data, supplementary information, acknowledgements, peer review information; details of author contributions and competing interests; and statements of data and code availability are available at 10.1038/s41592-022-01597-x.

## Supplementary information

Supplementary Information Supplementary Notes 1–4 and Supplementary Tables 1 and 2.

Reporting Summary

## Data Availability

DNA sequences of the sensors developed in this study have been deposited at DNA Data Bank of Japan (accession nos. LC720900–LC720924). Plasmid DNAs are available from either Addgene (plasmid nos. 184594–184620) or the corresponding author. The raw data for fluorescence recordings are not suitable for distribution through public repositories due to the large file size and are available from the corresponding author upon reasonable request. Source data are provided with this paper.

## Source data

Source Data Fig. 1 Statistical source data.
Source Data Fig. 2 Statistical source data.
Source Data Fig. 3 Statistical source data.
Source Data Fig. 4 Statistical source data.
Source Data Fig. 5 Statistical source data.
Source Data Fig. 6 Statistical source data.
Source Data Extended Data Fig. 1 Statistical source data.
Source Data Extended Data Fig. 3 Statistical source data.
Source Data Extended Data Fig. 4 Statistical source data.
Source Data Extended Data Fig. 7 Statistical source data.
Source Data Extended Data Fig. 8 Statistical source data.
Source Data Extended Data Fig. 9 Statistical source data.

## Code availability

Custom LabView codes used for data acquisition are available at https://github.com/daisukeino/MTRIAOT/.
