# Supplementary material for: A fluorescent sensor for real-time measurement of extracellular oxytocin dynamics in the brain
Source: Nat Methods. 2022 Sep 22;19(10):1286–94. doi: 10.1038/s41592-022-01597-x (PMC9550624; doi:10.1038/s41592-022-01597-x)
Supplement: Supplementary file 1 — Supplementary Notes 1–4 and Supplementary Tables 1 and 2. [file 41592_2022_1597_MOESM1_ESM.pdf]

nature portfolio

https://doi.org/10.1038/s41592-022-01597-x

Supplementary information

A fluorescent sensor for real-time measurement of extracellular oxytocin dynamics in the brain

In the format provided by the authors and unedited

Supplementary Note 1: Detailed description of sensor development

Optimization of the insertion site of cpGFP in the IL3 of meOTR

To engineer a fluorescent oxytocin (OT) sensor from the medaka oxytocin receptor (meOTR), we determined the optimal insertion site of circularly permutated green fluorescent protein (cpGFP) in the third intracellular loop (IL3) of meOTR (Extended Data Fig. 1b). Examination of 21 mutants expressed in human embryonic kidney 293T (HEK293T) cells, showed a fusion protein in which cpGFP was inserted into the region between K240 and I268 of meOTR (K240−I268) to give the best performance. This fusion protein had robust fluorescence, good plasma membrane targeting, and a slight fluorescence response ($\Delta F/F_0$ ~ 0.3) to 100 nM OT (Extended Data Fig. 1c−g). We therefore selected this chimera protein as the template for further engineering.

Screening of mutant sensors

To improve the fluorescence response of the initial OT sensor, we screened mutant sensors expressed in HEK293T cells using the following three steps (Fig. 1d). First, we optimized the linkers in the N- and C-terminal regions surrounding cpGFP. By examining the fluorescence responses of 127 mutants to 100 nM OT stimulation, we obtained OT-1.0, which had an approximately 150% $\Delta F/F_0$ response (Fig. 1e left). Next, we extended the mutagenesis to the neighboring regions, ranging from the transmembrane helix to the intracellular loops (TM-to-loop). Based on structural information of conformational transition during GPCR activation, we introduced mutations at sites from position 5.62 in the fifth transmembrane helix (S230 in meOTR) to position 6.36 in the sixth transmembrane helix (M278 in meOTR) (Extended Data Fig. 2). We examined 55 variants, of which five (S230C, F231Y, Q235R, L239G, and T274R in meOTR) showed increased fluorescence responses upon 100 nM OT stimulation. We then

developed an improved sensor, OT-2.0, which contained all five of these mutations. OT-2.0 had about a three-fold brighter basal fluorescence and larger fluorescence response (~390% $\Delta F/F_0$; Fig. 1e center). Finally, we further developed OT-2.0 by introducing a mutation within cpGFP. Based on previous knowledge of cpGFP mutagenesis, we screened 34 variants, of which three (S57T, S96M, and N202H in cpGFP; Extended Data Fig. 2b–c) had an increased rate of fluorescence change. We then constructed OT-3.0, a variant that contained all three of these mutations (Fig. 1e right).

Supplementary Note 2: Statistics in Figure 2

c, One-way ANOVA ($F_{5,24}$ = 2.62, P = 1.4 × 10^{-12}) with Dunnett's *post-hoc* test to compare with 0 (P = 1: 0.002 vs. 0, P = 1: 0.02 vs. 0, P = 0.0090: 0.2 vs. 0, P = 5.4 × 10^{-10}: 2 vs. 0, P = 5.2 × 10^{-14}: 20 vs. 0). **e**, One-way ANOVA ($F_{3,8}$ = 4.07, P = 0.001) with Tukey–Kramer *post-hoc* test (P = 0.002: ICV+OT vs. ICV+saline, P = 1: IN vs. ICV+saline, P = 1: IP vs. ICV+saline). **i**, One-way ANOVA ($F_{7,24}$ = 2.42, P = 3.3 × 10^{-6}) with Bonferroni *post-hoc* test (P = 0.02: 0 mW vs. 5 mW in ChRmine-mSca, P = 0.02: 0 mW vs. 12.5 mW in ChRmine-mSca, P = 0.002: 0 mW vs. 25 mW in ChRmine-mSca, P = 1: 0 mW vs. either 5 mW, 12.5 mW, or 25 mW in mSca).

Supplementary Note 3: Statistics in Figure 5

b, One-way ANOVA ($F_{2,8}$ = 4.46, P = 0.0016) with Bonferroni *post-hoc* test (P = 0.02: Before vs Mix-anes, P = 0.03: Mix-anes vs. Atipamezole). **d**, One-way ANOVA ($F_{2,12}$ = 3.89, P = 5.9 × 10^{-5}) with Bonferroni *post-hoc* test (P = 0.02: 1% vs 4%, P = 6.1 × 10^{-4}: 1% vs 0%: P = 0.014). **f**, One-way ANOVA ($F_{2,6}$ = 5.14, P = 0.0030) with Bonferroni *post-hoc* test (P = 0.019: Before vs. Food dep., P = 0.040: Food dep. vs. After, P = 1: before vs. after). **h**, One-way

ANOVA (top: $F_{3,8}$ = 4.06, P = 2.1 × 10^{-7}, bottom: $F_{3,8}$ = 4.07, P = 0.82) with Bonferroni *post-hoc* test (P = 0.1: 2 months vs 6 months, P = 0.0075: 2 months vs 1 year, P = 3.4 × 10^{-4}: 2 months vs 2.5 years in top graph).

Supplementary Note 4: Statistics in Extended Figure 3

b, Unpaired two-tailed t-test (P = 8.1 × 10^{-21}). **d**, One-way ANOVA ($F_{3,16}$ = 3.24, P = 0.0009) with Bonferroni *post-hoc* test (P = 0.029: (-) vs. (+) in meOTR-SmBit + LgBit-arrestin, P = 1: (-) vs. (+) in $MTRIA_{OT}$-SmBit + LgBit-arrestin). **e**, One-way ANOVA ($F_{4, 45}$ = 2.58, P = 7.0 × 10^{-22}) with Bonferroni post-hoc test (P = 9.7 × 10^{-13} Pre vs. 10 min, P = 1.9 × 10^{-13} Pre vs. 30 min, P = 9.3 × 10^{-15} Pre vs. 60 min, P = 2.6 × 10^{-11} Pre vs. 120 min, P = 0.24: 10 min vs. 30 min, P = 0.78: 10 min vs. 60 min, P = 1: 10 min vs. 120 min, P = 1: 30 min vs. 60 min, P = 1: 30 min vs. 120 min, P = 1: 60 min vs. 120 min). **g**, One-way ANOVA ($F_{4, 45}$ = 2.58, P = 0.063: top; $F_{4, 45}$ = 2.58, P = 1: bottom).

Supplementary Table 1: Basic properties of the MTRIA sensors.

Sensor	Receptor	Species	F_{max}	EC_{50} (M)
$MTRIA_{DA}$	Drd5	Medaka	2.40	9.31×10^{-7}
$MTRIA_{NE}$	Adrb2	Mouse	2.31	3.26×10^{-6}
$MTRIA_{5\text{-}HT}$	Htr1	Medaka	3.29	2.00×10^{-7}
$MTRIA_{Mtn}$	Mtnr1a	Medaka	4.06	6.11×10^{-9}
$MTRIA_{ATP}$	P2ry2	Mouse	1.27	3.04×10^{-6}
$MTRIA_{Agt}$	Agtr2	Medaka	2.84	3.60×10^{-8}
$MTRIA_{Avp}$	Avpr2	Medaka	2.46	2.00×10^{-8}
$MTRIA_{Cck}$	CckrL	Medaka	2.18	3.10×10^{-8}
$MTRIA_{Mch}$	Mchr1	Mouse	1.18	4.85×10^{-8}
$MTRIA_{Nmb}$	Nmbr	Zebrafish	2.13	4.93×10^{-9}
$MTRIA_{Npff}$	Npffr2	Mouse	1.73	7.28×10^{-8}
$MTRIA_{Npy}$	Npy2r	Medaka	2.29	6.13×10^{-8}
$MTRIA_{Nts}$	Ntsr1	Medaka	1.22	2.96×10^{-8}
$MTRIA_{Enk}$	Oprd1a	Zebrafish	1.70	1.18×10^{-8}
$MTRIA_{Ncp}$	Oprl1	Mouse	4.68	4.43×10^{-9}
$MTRIA_{Ox}$	Hcrtr2	Medaka	0.95	3.95×10^{-9}
$MTRIA_{Prlh}$	Prlhr2b	Zebrafish	0.72	2.75×10^{-8}
$MTRIA_{Sst}$	Sstr2	Medaka	2.50	5.39×10^{-8}
$MTRIA_{Nkb}$	Tac3r	Mouse	0.81	3.93×10^{-8}
$MTRIA_{Uts}$	Uts2r	Zebrafish	3.47	4.24×10^{-8}
$MTRIA_{C3a}$	C3ar1	Mouse	3.55	1.16×10^{-7}
$MTRIA_{Ltb}$	Ltb4r	Medaka	0.46	5.60×10^{-6}
$MTRIA_{Paf}$	Ptafr	Medaka	0.67	3.72×10^{-6}
$MTRIA_{Pge}$	Ptger4	Mouse	1.34	1.42×10^{-6}

Supplementary Table 2: GPCRs used in Fig. 6 and primer pairs for their cloning.

Ligand	Receptor (Best performance, Responded, Not-responded in Fig. 6)	Species	Primer F	Primer R
DA	DRD1	human	aaaGAATTCaccatgaagacgatcatcgcc	aaaGGATCCtcaggttgggtgctgaccg
	Drd1	mouse	aaaCTCGAGACCatggctcctaacacttctacc	tttACTAGTtcaggttgaatgctgtccgc
	Drd2	mouse	aaaCTCGAGACCatggatccactgaacctgtcc	tttACTAGTtcagcagtgcaggatcttcatg
	Drd3	mouse	aaaCTCGAGACCatggcacctctgagccagataag	tttACTAGTtcagcaggatagaatcttgagg
	Drd1	zebrafish	aaaGAATTCACCatggcagtaaaggatttgaatg	aaaGGATCCtcagttcttgtttaggcttgg
	Drd2	zebrafish	aaaGAATTCACCatggaagtcttcacagcgtatgc	aaaGGATCCtcaacagtgcagaatcttaatgaag
	Drd3	zebrafish	aaaGAATTCACCatggcaatgtttagcagtggtg	aaaGGATCCttagcagctcaggattttaatgaagg
	Drd6	zebrafish	aaaGAATTCACCatggagagcgatggtgctg	aaaGGATCCtcaacactcctcaatctgtccc
	Drd1	medaka	AAAGAATTCACCatggatctcatgaactttacaactgtaattg	aaaGGATCCtcatttcttgtttagactggctgtc
	Drd3	medaka	aaaGAATTCACCatggcgatgtttagcagcac	aaaGGATCCtcacgtagcccagccaggtg
	Drd5	medaka	aaaGAATTCACCatggagaatccagccaagtatc	aaaGGATCCtcagtgtaatccattgggtgtg
NE	ADRA2A	human	aaaGAATTCACCatggagacagacacactcc	aaaGGATCCtcacaacacgatccgcttcc
	Adra1	mouse	aaaGAATTCACCatggtgcttctttctgaaaatgcttc	aaaGGATCCtcatggataaaagccccggg
	Adrb1	mouse	aaaGAATTCACCatgggcgcgggggcgctc	aaaGGATCCctacaccttggactccgaggag
	Adrb2	mouse	aaaGAATTCACCatggggccacacgggaac	aaaACTAGTttacagtggcgagtcatttgtac
	Adra2a	zebrafish	aaaCTCGAGACCatgatttgtggggccaatgc	tttACTAGTtcacaccactctcctcttatctctc
	Adrb1	zebrafish	aaaCTCGAGACCatgggagacgggctaccg	tttACTAGTttacagctgagactctgaatggg
	Adrb2	zebrafish	aaaGAATTCACCatgggaaacataaggtcctcaatacc	aaaACTAGTttacaacactctcatttgggctttg
	Adrb3	zebrafish	aaaCTCGAGACCatggtgatcatcatcacgattacc	tttACTAGTtcagccatcctgctcctgaag
	Adra1	medaka	aaaGAATTCACCatgagtttgagcactaacaatgtcac	aaaGGATCCtcagaccttgtcatcgtttacaatg
	Adra2a	medaka	AAAGAATTCACCatggaggacagcaaccagaccag	aaaGGATCCtcacaggtacttcctccgc
	Adrb2	medaka	aaaGGATCCACCatggcaaatgagagttctgcg	aaaGCGGCCGCtcaaaccactgaagctttattttccg
ACh	CHRM3	human	aaaGAATTCACCatggagacagacacactcc	aaaGGATCCttacaaggcctgctcgggtg
	Chrm1	mouse	aaaGAATTCACCatgaacacctcagtgcccc	aaaGGATCCttagcattggcgggaggg
	Chrm4	mouse	aaaGAATTCACCatggcgaacttcacacctgtc	aaaGGATCCctacctggctgtgccgatg
	Chrm5	mouse	aaaCTCGAGACCatggaaggggagtcttatcacaatg	aaaACTAGTtcagggtagcttgctgttgc
	Chrm2	zebrafish	aaaGAATTCACCatggatacaataaacttcaccttctgg	aaaACTAGTtcatcgggtggagcgaatg
	Chrm5	zebrafish	aaaGAATTCACCatgggtgtggagaaattgaccc	aaaGGATCCtcatgtgagtttgctgccc
	Chrm3	medaka	AAAGAATTCACCatgagctccaacagtacagacc	aaaGGATCCttacgttgaatctctgggaatccg
	Chrm5	medaka	aaaGAATTCACCatggaaggagaaattgcgctaaact	aaaGGATCCtcatgacatcttggagctaaccac
His	Hrh1	mouse	aaaGAATTCACCatgagccttcccaacacctc	aaaGGATCCttaggaacgaatgtgcagaattttttg
	Hrh2	mouse	aaaGAATTCACCatggagcccaatggcacgg	aaaGGATCCttacctgactgggcttccctg
5-HT	Htr1	mouse	aaaGAATTCACCatggatatgttcagtcttggccag	aaaACTAGTtcagcggcagaacttgcac
	Htr7	mouse	aaaCTCGAGACCatgatggacgttaacagcagcg	aaaACTAGTtcacagttttgtcagtgcaacagaattc
	Htr6	zebrafish	aaaGAATTCACCatgcgtgttcgctcagagg	aaaACTAGTtcaatcatctaaagtatcgacctggttg
	Htr1	medaka	aaaGAATTCACCatggattttctaacattaagcggcaac	aaaACTAGTctatggtctgtgaaatttgcattttatgatc
	Htr4	medaka	aaaGAATTCACCatgaatgtcagcgctgcagg	aaaACTAGTctacacgggcactggtctg
	Htr5	medaka	aaaGAATTCACCatgacaagcaccaacgccag	aaaACTAGTtcaccgctgccgggaaaaaag
	Htr6	medaka	aaaGAATTCACCatgtctgagccccatctgc	aaaACTAGTtcaatccagattgtccgtctgattg
Mtn	Mtnr1a	mouse	aaaGAATTCACCatgaagggcaatgtcagcgag	gggACTAGTttaaacagagtccacctttattaagttattattg
	Mtnr1a	medaka	aaaGAATTCACCatgcttcaaaatgggtctcacc	aaaGGATCCtcagacggactccactttgac
	Mtnr1b	medaka	aaaGAATTCACCatgccggacgcaataaccctc	aaaGGATCCtcattccttgtttgtgcgatctc
	Mtnr1c	medaka	aaaGAATTCACCatggatttggaggtgaaggatg	aaaGGATCCttatacattgatctctgctacattgttg
ATP	P2ry2	mouse	aaaGAATTCACCatggcagcagacctggaac	aaaGGATCCctatagccgaatgtccttagtctcac
ADP	P2ry1	mouse	aaaGAATTCACCatgaccgaggtgccttgg	aaaGGATCCtcacaaactcgtgtctccattctg
	P2ry12	mouse	aaaGAATTCACCatggatgtgcctggtgtcaac	aaaGGATCCctacattggggtctcttcgcttg
	P2ry1	zebrafish	aaaGAATTCACCatgacagcggagtttaataacctg	aaaGGATCCtcacatgcggttttcaccg
	P2ry12	zebrafish	aaaGAATTCACCatggagcaaacaacgcagc	aaaACTAGTtcatgtcagtgcgtttccctg
	P2ry1	medaka	aaaGAATTCACCatgaccacagacctgaacttgac	aaaACTAGTtcacagtctgcgctccc
	P2ry12	medaka	aaaGAATTCACCatggacttaaatgccactctgttc	aaaGGATCCtcaattacatgtagacatttgttggc
UDP	P2ry6	mouse	aaaGAATTCACCatggagcaggacaatggcac	aaaGGATCCtcagactctctgcctctgcc
	P2ry6	medaka	aaaGAATTCACCatgccccccatcgtccaac	aaaGGATCCtcaggactttgacacagcgg
Ado	Adora1	mouse	aaaGAATTCACCatgccgccgtacatctcg	aaaGGATCCctagtcatcagctttctcctctgg
	Adora2a	mouse	tttAAGCTTACCatgggctcctcggtgtac	tttACTAGTtcaggaaggggcaaactctg
	Adora2b	mouse	aaaGAATTCACCatgcagctagagacgcaagac	aaaGGATCCtcataagcccagactgagagtag
	Adora3	mouse	aaaGAATTCACCatggaagccgacaacaccac	tttACTAGTttactcagtagtctgttccatgttg
	Adora2a	zebrafish	aaaGAATTCACCatgctgaacaatgttttcgacgtg	aaaGGATCCtcaggaaacctccgtgagttc
	Adora2b	zebrafish	aaaGAATTCACCatggattcgctctacatcgcc	aaaGGATCCctatagcagagggtcaatagtcattg
	Adoa2b	medaka	aaaGAATTCACCatgagtcctgaaacggaccag	aaaGGATCCttacagcagagggtcgatggtc
Agt	Agtr2	mouse	aaaGAATTCACCatgaaggacaacttcagttttgctg	aaaGGATCCttaagacacaaaggtgtccatttctc
	Agtr2	zebrafish	aaaGAATTCACCatggaaccagactccgcc	aaaGGATCCttacgagctctgctgatccag
	Agtr2	medaka	aaaGAATTCACCatgatggcaatcccaactgac	aaaGGATCCctacgagggattagaagtctccac
Avp	Avpr1a	mouse	aaaGAATTCACCatgagtttcccgcgaggc	aaaGGATCCtcaagtggagacagggataaatc

	Avpr1b	mouse	aaaGAATTCACCatggattctgagccttcttg	aaaGGATCCctaagagatgctggtctcc
	Avpr2	mouse	aaaGAATTCACCatgatcctggtgtctaccac	aaaGGATCCtcagagaggagctggctg
	Avpr1a	zebrafish	aaaGAATTCACCatggagacgcacagtaacacg	aaaGGATCCtcatgtgttggccggcgtg
	Avpr1b	zebrafish	aaaGAATTCACCatgggcaacacgtcgaacc	aaaGGATCCctaggactccataggcacgc
	Avpr2	zebrafish	aaaCTCGAGACCatggagaccttctcaagagag	aaaACTAGTtcagtactggttgtccttgg
	Avpr1a	medaka	aaaCTCGAGACCatgctcttcccgtcggacagc	aaaGGATCCtcagctttctgctcgtgacaaatc
	Avpr1b	medaka	aaaGAATTCACCatgtacactctctccagcgtgc	aaaGGATCCttactctgcctgagcagagtttttg
	Avpr2	medaka	AAAGAATTCACCatggaaagcatcaatgtggagag	tttGCGGCCGCtcagtataggttgtccttggtgg
Bdk	Bdkrb2	mouse	aaaGAATTCACCatgccctgctcctggaag	aaaGGATCCtcactgtttcttccctgccc
	Bdkrb1	zebrafish	aaaCTCGAGACCatgcaaccagaagagtttacatcatc	aaaGGATCCctagtctttttcgttcagaaccacag
	Bdkrb2	medaka	aaaGAATTCACCatgacttttcagcccacaagtttc	aaaGGATCCctagaccaaactcttgagcgttg
Cck	Cckbr	mouse	aaaGAATTCACCatggatctgctcaagctgaacc	aaaACTAGTtcagccaggtcccagcgtgc
	Cckar	zebrafish	aaaGAATTCACCatggagacatttacaattcaagatatgctc	aaaGGATCCctatgcttgtgctgaaccacg
	Cckar	medaka	aaaGAATTCACCatgggggagtcgtttaccacc	aaaGGATCCtcagttgtaggtaaagcgagtgctc
	CckrL	medaka	aaaGAATTCACCatggatactttgagaaacgag	tttACTAGTtcagcagtttcccatggtgc
Crh	Crhr1	mouse	aaaGAATTCACCatgggacagcgcccgcagctc	aaaGGATCCtcacactgctgtggactgcttg
	Crhr1	zebrafish	aaaGAATTCACCatgagtcgcatcctccatccac	aaaACTAGTtcagacggctgacgactgcttg
	Crhr2	medaka	aaaCTCGAGACCatgcgctcccgggatgcgcg	aaaGGATCCtcacaccgctgtggtctgc
ET-1	Edra	mouse	aaaCTCGAGACCatgagtatcttttgccttgcg	aaaACTAGTttagttcatgctgtccttgtgg
	Ednrb	mouse	aaaGAATTCACCatgcaatcgcccgcaagc	aaaACTAGTtcaagacgagctgtatttattgctg
	Ednra	medaka	aaaGAATTCACCatggccccaccagcagg	aaaGGATCCtcagttgctgtctttcctgaag
	Ednr	medaka	aaaGAATTCACCatgagggccagtgtgctg	aaaACTAGTttaggaggagctggtgtatttctg
Gal	Galr1	mouse	aaaGAATTCACCatggaactggctatggtgaacc	aaaGGATCCtcacacgtgggtgcagttg
	Galr1a	medaka	aaaGAATTCACCatgaacttgtcggagtctctgtg	aaaGGATCCtcaaacattagtgcaattagttgaggc
	Galr1b	medaka	aaaGAATTCACCatgctgccaggaaacgactc	aaaGGATCCtcacacattggtggaagagtgtg
	Galr2	medaka	aaaGAATTCACCatggctgatttcgaggatttc	aaaGGATCCtcaagtcggttgctgaaacggc
Ghr	Ghra	mouse	aaaGAATTCACCatgtggaacgcgacgcc	aaaGGATCCtcatgtattgatgctcgactttgtcc
	Ghra	zebrafish	aaaGAATTCACCatgcctacctggacgaaccg	aaaGGATCCtcacaggctggctgtagattc
Msh	Mc1r	mouse	aaaGAATTCACCatgtccactcaggagccccag	aaaGGATCCtcaccaggagcacagcagcac
	Mc3r	mouse	aaaGAATTCACCatgaactcttcctgctgcctg	aaaGGATCCctagcccaagttcatgctgttg
	Mc4r	mouse	aaaGAATTCACCatgaactccacccaccaccatg	aaaGGATCCttaatacctgctagacaactcacagatg
	Mc5r	mouse	aaaGAATTCACCatgaactcctcctccaccctg	aaaGGATCCttaatacccgccaaggagcctac
	Mc1r	zebrafish	aaaGAATTCACCatgaacgactcttcgcgccatc	aaaGGATCCttacactgcaaagcaccacgaac
	Mc3r	zebrafish	aaaGAATTCACCatgaacgactcacatctgcagtttc	aaaGGATCCctaaagtgcaggctggcagcc
	Mc1r	medaka	aaaGAATTCACCatggccaacagctcgttccac	aaaGGATCCtcacacgctgaagcagaaggaac
	Mc4r	medaka	aaaGAATTCACCatgaactccactctgccttatggg	aaaGGATCCtcacacacacaggagagcgttg
	Mc5r	medaka	aaaGAATTCACCatggaagtgacagataaaaccaattctttg	aaaGGATCCttagtacttaccggtcagagcacag
Mch	Mchr1	mouse	aaaGAATTCACCatggatctgcaagcctcgttg	aaaGGATCCtcaggtgcctttgctttctgtc
	Mchr2	medaka	aaaGAATTCACCatggatcccatcatgaatgacac	aaaACTAGTtcaaatcacggttatgcgatagttg
Nmb	Nmbr	mouse	aaaGAATTCACCatgccccccaggtctctc	aaaGGATCCtcacagtgctatttcttgctttgtg
	Nmbr	zebrafish	aaaGAATTCACCatggatcatactttctccgaggatac	aaaGGATCCctaaatgcagactccttgtttttggc
	Nmbr	medaka	aaaGAATTCACCatggatgacgagttccctcc	aaaGGATCCtcacagcgccgcttcctg
Nmu	Nmur	mouse	aaaGAATTCACCatgactcctccctgcctc	aaaACTAGTtcaggaggggtctgtctc
	Nmur	medaka	aaaGAATTCACCatgtcagctgccaactgctc	aaaGGATCCctaatattttttgattcagtaatctcgtctttc
Npff	Npffr1	mouse	aaaGAATTCACCatggaggcggaaccctccc	aaaGGATCCtcaaatgttccaggctgggatag
	Npffr2	mouse	aaaGAATTCACCatgagcgagaaatgggactcaaac	aaaGGATCCttaagccacagtactgttagtagcttc
	Npffr2	medaka	aaaGAATTCACCatgaacgaaagcctggagaacaac	aaaGGATCCtcagatggacacggccgtc
Npy	Npy2r	mouse	aaaGAATTCACCatgggcccggtaggtgc	aaaGGATCCttacacattggtagcctccgaaaaag
	Npy5r	mouse	aaaGAATTCACCatggaggttaaacttgaagagcatttt	aaaGGATCCtcatgacatgtgtaggcagtgg
	Npy1r	zebrafish	aaaGAATTCACCatgccagactccgccttc	aaaGGATCCtcagagatctaggctactcatttttaac
	Npy8r	zebrafish	aaaGAATTCACCatggaggccaacatcactaacatc	aaaGGATCCtcagcagtgagcgcattg
	Npy2r	medaka	aaaGAATTCACCatggatccagaagatcaactaaacatg	aaaACTAGTttagacgtttgtcgatttatcatcagtac
	Npy8r	medaka	aaaGAATTCACCatgcccaacagcagcggc	aaaGGATCCtcagacgttggctggtagag
Nts	Ntsr1	mouse	aaaGGATCCctagtacagggtttcccggg	aaaGGATCCctagtacagggtttcccggg
	Ntsr1	medaka	aaaGAATTCACCatggatgtgaactcctcgctg	aaaGGATCCtcagtatgctgtttctttaatcatgtg
Enk	Oprd1	mouse	aaaGAATTCACCatggagctggtgccctctg	aaaGGATCCtcaggcggcagcgccacc
	Oprd1a	zebrafish	aaaCTCGAGACCatggagccgtccgtcattcc	aaaGAATTCtcataccggcttctttcctgtatcc
	Oprd1b	zebrafish	aaaGAATTCACCatggagcctccaacagtgactg	aaaGGATCCtcatgtgggctgcttgattgattc
	Oprd1	medaka	aaaCTCGAGACCatggaaaatactcctgttgaaatttttaaag	aaaGCGGCCGCtcatgttggccgactagtcc
Noc	Oprl1	mouse	aaaGAATTCACCatggagtccctctttcctgc	aaaGGATCCtcatgccggccgtggtac
	Oprl1	zebrafish	aaaGAATTCACCatggagttcccaaatgattccatc	aaaACTAGTtcatgcgggattactgttccc
Ox	Hctr2	medaka	aaaGAATTCACCatgtctggaatctctgggaatttg	aaaGGATCCtcaagacaaaacgatctgctctgac
Prlh	Prlhr2a	zebrafish	aaaCTCGAGACCatggatggctgtggtggag	aaaACTAGTtcaaagaaccacgctagcgg
	Prlhr2b	zebrafish	aaaGAATTCACCatggaggcctctggttgg	aaaGGATCCtcatagtacaacgctggccg
	Prlhr2l	medaka	aaaGAATTCACCatggactggaaaagcgcg	aaaGGATCCctagctgtagttttcctggggaatc
Pth	Pth1r	mouse	aaaGAATTCACCatggggaccgcccggatc	aaaGGATCCtcacatgactgtttcccattcttc
	Pth2r	mouse	aaaGAATTCACCatggcctggctggagactttc	aaaACTAGTtcagataggatgggtttctcccttg
	Pth1r	medaka	aaaGAATTCACCatgggatcctctcagctgc	aaaACTAGTtcacatgactgtctcccgc

Sst	Sstr2	mouse	aaaGAATTCACCatggagatgagctctgagcag	aaaGGATCCtcagatactggtttggaggtctc
	Sstr3	mouse	aaaCTCGAGACCatggccactgttacctatccttc	aaaGAATTCttacagatggctcagtgtgctg
	Sstr4	mouse	aaaGAATTCACCatgaacgcgccagcaactc	aaaGGATCCtcagaaagtagtggtcttggtgaaag
	Sstr1	zebrafish	aaaGAATTCACCatgctgcccaacgacacc	aaaGGATCCctatagtgttgtagttctggatgtgc
	Sstr1	medaka	aaaGAATTCACCatgatttatatgcagaacattcaagggg	aaaGGATCCttacagtgtagttgtccgggagg
	Sstr2	medaka	aaaGAATTCACCatggagtcctgggcttttcc	aaaGGATCCttagatgcttgtctgcaggtctc
SP	Tacr1	zebrafish	aaaGAATTCACCatggattcgttcatcacttccac	aaaACTAGTtcattcctgtaggttattactggagtag
	Tacr1	medaka	aaaGAATTCACCatggatcctctgtttaatacaagcg	aaaACTAGTttattcctgtgtgttgtaactgg
Nkb	Tac3r	mouse	aaaGAATTCACCatggcctcggttcccacc	aaaGGATCCttaggaatattcatccacagaggtatagg
	Tac3r	medaka	aaaGAATTCACCatgggagccccgaataacg	aaaACTAGTtcaagagaactcctcaggctc
Uts	Uts2r	mouse	aaaGAATTCACCatggcgctgagcctggag	aaaGGATCCtcacacaaaggccccattagg
	Uts2r	zebrafish	aaaGAATTCACCatgctaaacgtctgtctgcttg	aaaACTAGTtcagaggctgctgttgttgg
C3a	C3ar1	mouse	aaaGAATTCACCatggagtctttcgatgctgacac	aaaGGATCCtcacacatctgtactcatattgtttc
	C3ar1	medaka	aaaCTCGAGACCatgaatggaacagggctaaatgtg	aaaGGATCCctacacctgggagttcttgatagac
	C3ar2	medaka	aaaGAATTCACCatgatggtctccaacatctccc	aaaACTAGTttaaatctttgctacgtccagactc
C5a	C5ar1	mouse	aaaGAATTCACCatggaccccatagataacagcag	aaaACTAGTctacaccgcctgactcttcc
	C5ar2	mouse	aaaCTCGAGACCatgatgaaccacaccaccag	aaaGAATTCctacaccggcatctcagacac
	C5ar1	medaka	aaaGAATTCACCatggaaaacatgtatgattatactaacacaag	aaaGGATCCctacactttggtcgtccccttc
Thr	F2r	mouse	aaaGAATTCACCatggggccccggcgcttg	aaaGGATCCctaagctaatagctttttgtatatgctg
	F2r	medaka	aaaCTCGAGACCatgtggacggcgtgcagc	aaaGAATTCtcaggcctccagcctgctg
Ana	Cnr1	mouse	aaaGAATTCACCatgaagtcgatcttagacggcc	aaaGGATCCtcacagagcctcggcagac
	Cnr2	medaka	aaaCTCGAGACCatggaccccagttctggatcg	tttACTAGTtcaatttttcccatcctgctgac
S1P	S1pr1	mouse	aaaGAATTCACCatggtgtccactagcatccc	aaaGGATCCttaggaagaagaattgacgtttcc
	S1pr2	mouse	aaaGAATTCACCatgggcggcttatactcagag	aaaGGATCCtcagaccactgtgttaccctcc
	S1pr3	mouse	aaaGAATTCACCatggcaaccacgcatgcg	aaaGGATCCtcacttgcagaggaccccg
	S1pr4	mouse	aaaGAATTCACCatgaacatcagtacctggtccac	aaaACTAGTctaggtgctgcggacgc
	S1pr5	mouse	aaaGAATTCACCatggagcccgggctgctg	aaaGGATCCtcagtctgtagcagtaggcac
	S1pr1	medaka	aaaGAATTCACCatgacggcttcaagttctcttc	aaaGGATCCctatgacttgaagagcttgggatatatag
	S1pr2	medaka	aaaGAATTCACCatgagtctttcccatagtgccc	aaaGGATCCtcagacacaggtggtacagtc
	S1pr3	medaka	aaaGAATTCACCatggggcgcatcatggaag	aaaGGATCCttagaacttgttgagggctggc
Ltb	Ltb4r2	mouse	aaaGAATTCACCatgtctgtctgctaccgtcc	aaaGGATCCctaccattcttgactgtctttctcc
	Ltb4r2a	zebrafish	aaaGAATTCACCatggcccttcagagtccatc	aaaGGATCCtcactttccattattctggggtgc
	Ltb4r2b	zebrafish	aaaGAATTCACCatggcattggaaaatggcagc	aaaGGATCCttatagcctgatgacatccctagtctg
	Ltb4r	medaka	aaaGAATTCACCatggcatccaccggaaacatc	aaaGGATCCttattcacactgttcaacagtggc
	Ltb4r1	medaka	aaaGAATTCACCatggagcaactcaacttgactg	aaaGGATCCtcattggtttagatggttcacacatttac
	Ltb4r2	medaka	aaaGAATTCACCatgaagaaaaataccacagttcacgag	aaaGGATCCctaactgacttcaggagttgctg
Lte	Cysltr2	mouse	aaaCTCGAGACCatggaagtaactgggacccc	aaaACTAGTctatagatgaactttgctgaatatagccc
	Gpr17	mouse	aaaCTCGAGACCatgaacggtctggaggcag	aaaACTAGTtcacagctcggatcggg
	Cysltr1	zebrafish	aaaCTCGAGACCatggctaacttaacagactgccc	aaaACTAGTctacccatgtgtgtttgaaccattac
Lpa	Lpar1	mouse	aaaGAATTCACCatgaacgaacaacagtgcttctac	aaaGGATCCctaaaccacagagtggtcgttg
	Lpar3	medaka	aaaGAATTCACCatggaccagcagaaccaaaacc	aaaGGATCCtcactccatttcatctgatggtagc
Paf	Ptafr	mouse	aaaGAATTCACCatggagcacaatggctcctttc	aaaGGATCCttaatttttcagcgacacaataggagtc
	Ptafr	medaka	aaaCTCGAGACCatggagggaaccacagaccaag	aaaACTAGTtcacgattgattgggttgttctaag
Pgd	Ptgdr	mouse	aaaGAATTCACCatgaacgagtcctatcgctgtc	aaaGGATCCtcacaaagtggattccacgttac
Pge	Ptger2	mouse	aaaGAATTCACCatggacaattttcttaatgactccaagc	aaaGGATCCtcacaactgtccacaaaggtcag
	Ptger3	mouse	aaaGAATTCACCatggctagcatgtgggcg	aaaACTAGTtcatctttccagctggtcactc
	Ptger4	mouse	aaaGAATTCACCatgtccatccccggagtc	aaaGGATCCctatatacatttttcagataatttcagagtttcactgg
	Ptger1	zebrafish	aaaGAATTCACCatgttatccatccagcaatacaacag	aaaGGATCCtcaggtctgattaatggctttcttc
	Ptger3	zebrafish	aaaGAATTCACCatggcatcaaaatgtaagtcttcaac	aaaACTAGTtcactgttcttcacatcccacg
	Ptger2	medaka	aaaCTCGAGACCatgctgaatgactcatgccac	aaaACTAGTtcaaatgttttcccgagaagtaaagtc
Pgf	Ptgfr	mouse	aaaCTCGAGACCatgtctatgaacagttccaagcag	aaaACTAGTttacagtccagcttcactcgatg
Pgi	Ptgir	mouse	aaaGAATTCACCatgatggccagcgatggac	aaaGGATCCtcagcagagggagcagg
	Ptgir	medaka	aaaCTCGAGACCatgaccaacagcagtaactgc	aaaACTAGTtcacgccaaggggctg
