# Supplementary material for: A fluorescent sensor for real-time measurement of extracellular oxytocin dynamics in the brain
Source: Nat Methods. 2022 Sep 22;19(10):1286–94. doi: 10.1038/s41592-022-01597-x (PMC9550624; doi:10.1038/s41592-022-01597-x)
Supplement: Supplementary file 2 — Reporting Summary [file 41592_2022_1597_MOESM2_ESM.pdf]

nature portfolio

Corresponding author(s): Daisuke Ino

Last updated by author(s): May/29/2022

Reporting Summary

Nature Portfolio wishes to improve the reproducibility of the work that we publish. This form provides structure for consistency and transparency in reporting. For further information on Nature Portfolio policies, see our Editorial Policies and the Editorial Policy Checklist.

Statistics

For all statistical analyses, confirm that the following items are present in the figure legend, table legend, main text, or Methods section.

n/a	Confirmed	
☐	☒	The exact sample size (*n*) for each experimental group/condition, given as a discrete number and unit of measurement
☐	☒	A statement on whether measurements were taken from distinct samples or whether the same sample was measured repeatedly
☐	☒	The statistical test(s) used AND whether they are one- or two-sided<br>*Only common tests should be described solely by name; describe more complex techniques in the Methods section.*
☒	☐	A description of all covariates tested
☐	☒	A description of any assumptions or corrections, such as tests of normality and adjustment for multiple comparisons
☐	☒	A full description of the statistical parameters including central tendency (e.g. means) or other basic estimates (e.g. regression coefficient) AND variation (e.g. standard deviation) or associated estimates of uncertainty (e.g. confidence intervals)
☐	☒	For null hypothesis testing, the test statistic (e.g. *F*, *t*, *r*) with confidence intervals, effect sizes, degrees of freedom and *P* value noted<br>*Give P values as exact values whenever suitable.*
☒	☐	For Bayesian analysis, information on the choice of priors and Markov chain Monte Carlo settings
☒	☐	For hierarchical and complex designs, identification of the appropriate level for tests and full reporting of outcomes
☐	☒	Estimates of effect sizes (e.g. Cohen's *d*, Pearson's *r*), indicating how they were calculated

Our web collection on statistics for biologists contains articles on many of the points above.

Software and code

Policy information about availability of computer code

Data collection	Fusion software (Andor, ver. 2.1) and cellSense (Olympus, ver. 2.2) was used for data acquisition in spinning-disk confocal microscopic analyses.Custom-designed LabVIEW software (National Instruments, ver. 2018 or 2020) was used for data acquisition in fiber photometry analyses.
Data analysis	ImageJ software (NIH, ver. 1.52) and python modules (numpy, scipy.optimize.curve_fit) were used for image analysis and data processing. R (ver. 4.05) and Excel (Microsoft, 2016 or 2019) were used for statistical analysis and data plotting.

For manuscripts utilizing custom algorithms or software that are central to the research but not yet described in published literature, software must be made available to editors and reviewers. We strongly encourage code deposition in a community repository (e.g. GitHub). See the Nature Portfolio guidelines for submitting code & software for further information.

Data

Policy information about availability of data

All manuscripts must include a data availability statement. This statement should provide the following information, where applicable:

- Accession codes, unique identifiers, or web links for publicly available datasets
- A description of any restrictions on data availability
- For clinical datasets or third party data, please ensure that the statement adheres to our policy

Plasmid DNAs are available either from Addgene (plasmid #184594–184620) or the corresponding author. Source data are provided with this paper. All other raw data can be made available upon reasonable request.

Field-specific reporting

Please select the one below that is the best fit for your research. If you are not sure, read the appropriate sections before making your selection.

☒ Life sciences ☐ Behavioural & social sciences ☐ Ecological, evolutionary & environmental sciences

For a reference copy of the document with all sections, see nature.com/documents/nr-reporting-summary-flat.pdf

Life sciences study design

All studies must disclose on these points even when the disclosure is negative.

Sample size	No statistical methods were used to predetermine the sample sizes, but our sample sizes were similar to those generally used in the field (Sun et al., Cell 2018; Patriarchi et al., Science 2018; Feng et al., Neuron 2019; Jing et al., Nat Methods 2020; Peng et al., Science 2020).
Data exclusions	For fiber photometry analysis, mice with misplacement of the cannula were excluded from the analysis.
Replication	All attempts at replication were successful. All experiments were repeated at least twice.
Randomization	Cells and animals were randomly assigned into control or experimental groups.
Blinding	Data collection and analysis were not performed in a blinded manner because the experimental conditions were obvious to the researchers. However, standardized procedures for data collection and analysis were used to prevent human bias.

Reporting for specific materials, systems and methods

We require information from authors about some types of materials, experimental systems and methods used in many studies. Here, indicate whether each material, system or method listed is relevant to your study. If you are not sure if a list item applies to your research, read the appropriate section before selecting a response.

Materials & experimental systems

n/a	Involved in the study	
☐	☒	Antibodies
☐	☒	Eukaryotic cell lines
☒	☐	Palaeontology and archaeology
☐	☒	Animals and other organisms
☒	☐	Human research participants
☒	☐	Clinical data
☒	☐	Dual use research of concern

Methods

n/a	Involved in the study	
☒	☐	ChIP-seq
☒	☐	Flow cytometry
☒	☐	MRI-based neuroimaging

Antibodies

Antibodies used	Rabbit anti-HA polyclonal antibody (MBL, 561, 1:2000 dilution), rabbit anti-GFP polyclonal antibody (MBL, 598, 1:1000 dilution), rabbit anti-OT monoclonal antibody (abcam, ab212193, 1:2000 dilution), and Alexa 488-conjugated goat anti-rabbit IgG antibody (Jackson ImmunoResearch, 111-545-144, 1:4000 dilution for ICC and 1:2000 dilution for IHC) were used in this study.
Validation	The antibodies listed above have been validated in the previous reports: anti-HA (https://ruo.mbl.co.jp/bio/dtl/A/index.html?pcd=561), anti-GFP (https://ruo.mbl.co.jp/bio/dtl/A/index.html?pcd=598), anti-OT (https://www.abcam.com/oxytocin-antibody-epr20973-ab212193.html), and Alexa 488-conjugated anti-rabbit IgG (https://www.jacksonimmuno.com/catalog/products/111-545-144).

Eukaryotic cell lines

Policy information about cell lines

Cell line source(s)	293T cells (ATCC)
Authentication	The cell line used has not been authenticated, but its morphology was similar to that reported by the supplier (https://www.atcc.org/products/crl-3216).
Mycoplasma contamination	Cell lines used were not tested for mycoplasma contamination.
Commonly misidentified lines (See ICLAC register)	No commonly misidentified cell lines were used.

Animals and other organisms

Policy information about studies involving animals; ARRIVE guidelines recommended for reporting animal research

Laboratory animals	C57BL/6 J or C57BL/6N female mice at either 6–8 weeks postnatal, 6 months postnatal, 1 year postnatal or 2.5 years postnatal were purchased from CLEA Japan Inc., and were used in this study.
Wild animals	No wild animals were used in the study.
Field-collected samples	No field-collected samples were used in the study.
Ethics oversight	The animal experiments were reviewed and approved by Institutional Animal Use and Care Committees of Kanazawa University and Osaka University.

Note that full information on the approval of the study protocol must also be provided in the manuscript.
